# Dementia risk in Parkinson’s disease is associated with interhemispheric connectivity loss and determined by regional gene expression

**DOI:** 10.1016/j.nicl.2020.102470

**Published:** 2020-10-15

**Authors:** Angeliki Zarkali, Peter McColgan, Mina Ryten, Regina H. Reynolds, Louise-Ann Leyland, Andrew J. Lees, Geraint Rees, Rimona S. Weil

**Affiliations:** a Dementia Research Centre, University College London, 8-11 Queen Square, London WC1N 3AR, UK; b Huntington’s Disease Centre, University College London, Russell Square House, London WC1B 5EH, UK; c NIHR Great Ormond Street Hospital Biomedical Research Centre, University College London, London, UK; d Great Ormond Street Institute of Child Health, Genetics and Genomic Medicine, University College London, London, UK; e Department of Neurodegenerative Disease, UCL Institute of Neurology, 10-12 Russell Square House, London WC1B 5EH, UK; f Reta Lila Weston Institute of Neurological Studies, 1 Wakefield Street, London WC1N 1PJ, UK; g Institute of Cognitive Neuroscience, University College London, 17-19 Queen Square, London WC1N 3AR, UK; h Wellcome Centre for Human Neuroimaging, University College London, 12 Queen Square, London WC1N 3AR, UK; i Movement Disorders Consortium, University College London, London WC1N 3BG, UK

**Keywords:** Parkinson’s disease, Parkinson’s disease dementia, Diffusion weighted imaging, Connectomics, White matter, Regional gene expression

## Abstract

Parkinson’s dementia is a common and devastating part of Parkinson’s disease. Whilst timing and severity vary, dementia in Parkinson’s is often preceded by visual dysfunction. White matter changes, representing axonal loss, occur early in the disease process. Clarifying which white matter connections are affected in Parkinson’s with visual dysfunction and why specific connections are vulnerable will provide important mechanistic insights. Here, we use diffusion tractography in 100 Parkinson’s patients (33 low visual performers) and 34 controls to identify patterns of connectivity loss in Parkinson’s with visual dysfunction. We examine the relationship between regional transcription and connectivity loss, using the Allen Institute for Brain Science transcriptome atlas. We show that interhemispheric connections are preferentially affected in Parkinson’s low visual performers. Interhemispheric connection loss was associated with downweighted genes related to the smoothened signalling pathway (enriched in glutamatergic neurons) and upweighted metabolic genes. Risk genes for Parkinson’s but not Alzheimer’s or Dementia with Lewy bodies were over-represented in upweighted genes associated with interhemispheric connection loss. Our findings suggest selective vulnerability in Parkinson’s patients at highest risk of dementia (those with visual dysfunction), where differences in gene expression and metabolic dysfunction, affecting longer connections with higher metabolic burden, drive connectivity loss.

## Highlights

• Dementia risk in PD is associated with interhemispheric structural connectivity loss.
• Connection type rather than length alone determines selective vulnerability of connections.
• Specific regional gene expression patterns and cell types are associated with interhemispheric connection loss.

## 1 Introduction

Dementia is a common and disabling symptom in Parkinson’s disease, but the structural changes at the early stages are not yet known. Parkinson’s patients with visual dysfunction are at higher risk of Parkinson’s dementia: PD patients who make errors copying intersecting pentagons show double dementia rates (Williams-Gray et al., 2013). Poor colour vision predicts PD dementia (Anang et al., 2014) and impaired visual performance in higher-order visual tasks is strongly correlated with deteriorating cognition at follow-up (Weil et al., 2019). Therefore, Parkinson’s patients with visual dysfunction are a useful group to identify early structural changes linked with Parkinson’s dementia.

Animal and cellular models of Parkinson’s disease show that the earliest degenerative changes involve alpha-synuclein accumulation in the axonal compartment (Chung et al., 2009, Prots et al., 2018). Exogenous alpha-synuclein in neuronal cultures leads to formation of endogenous pathology that starts within the axon (Volpicelli-Daley et al., 2011). Mouse models for leucine rich repeat kinase 2 (LRRK2) gene, the commonest genetic cause of PD, exhibit axonal pathology before neurite loss (Li et al., 2009). Therefore in vivo measures of white matter integrity in Parkinson’s patients with visual dysfunction are likely to provide important mechanistic insights for the early stages of Parkinson’s dementia.

Diffusion imaging can detect white matter (WM) alterations early in patients with PD, even in the absence of other imaging changes (Agosta et al., 2014, Duncan et al., 2016). We recently showed that PD patients with visual impairment, who are at risk of dementia but cognitively intact, also exhibit WM changes, particularly in interhemispheric connections (Zarkali et al., 2020). Brain networks exhibit small-world topology, favouring clusters of highly connected regions with relatively few high-cost, long-range connections (Bassett and Bullmore, 2006, Laughlin and Sejnowski, 2003). These long connections are critical for whole network integration (van den Heuvel and Sporns, 2013). One such important connection type are interhemispheric connections, which are preferentially affected in schizophrenia (Chang et al., 2019, Grazioplene et al., 2018), and neurodegenerative disorders such as Huntington’s disease (McColgan et al., 2017).

The underlying pathological processes that determine which connection types are most affected in PD are not yet known, but differences in regional gene expression may drive selective vulnerability for particular connections. Regional gene expression in health is linked with WM loss in Huntington’s disease (McColgan et al., 2018) and schizophrenia (Romme et al., 2017). In PD, differential expression of predetermined candidate genes has been associated with cortical atrophy (Freeze et al., 2019, Freeze et al., 2018). However, grey matter atrophy is variable and occurs relatively late in PD with cognitive impairment (Agosta et al., 2014, Hanganu et al., 2014, Melzer et al., 2012, Song et al., 2011). The regional transcriptome patterns associated with WM connectivity loss may provide insights into the selective vulnerabilities underlying the early stages of cognitive impairment in PD.

Here we aimed to clarify the patterns of WM connectivity loss in PD patients with low visual performance, who are at higher risk of developing dementia, and shed light onto the pathological processes that drive this loss. We first classified WM connections into subtypes: connections between cortical modules and subcortical structures (subcortical-cortical), between cerebral hemispheres (interhemispheric), within hemispheres (intrahemispheric), and within cortical modules (intramodular). We examined how these connections differ in PD low visual performers and show that connection type determines vulnerability. Next, we investigated two potential drivers of selective vulnerability: connection length and regional gene expression. We calculated connection length in control participants to examine whether connections that are normally longer are more vulnerable to connectivity loss PD. Finally, we examined how differences in regional gene expression in the healthy brain are associated with WM vulnerability in patients with PD and visual dysfunction and whether different biological processes determine this WM loss.

## 2 Materials and methods

### 2.1 Participants

100 patients with Parkinson’s disease (PD) and 34 unaffected controls were included (Table 1). All patients satisfied the Queen Square Brain Bank criteria for PD (Daniel and Lees, 1993). The study was approved by the local ethics committee and participants provided informed consent. Whole brain fixel based analysis of this cohort has been previously published (Zarkali et al., 2020).

**Table 1:** Demographics and clinical characteristics in controls, PD high visual performers and PD low visual performers.

Characteristic	Controls n = 34	PD high visual performers n = 67	PD low visual performers n = 33	Statistic
Demographics				
Age (years)	66.4 (9.3)	**62.2 (7.2)**	**68.7 (7.3)**	**t = 4.22** **p < 0.001**
Male (%)	16 (45.7)	32 (47.8)	21 (63.6)	x^2^ = 2.26 p = 0.135
Total intracranial volume (ml)	1397.3 (106.4)	1462.6 (124.8)	1469.4 (139.1)	t = 0.25 p = 0.805
Mood				
HADS anxiety	3.8 (3.5)	5.9 (3.8)	6.2 (4.7)	U = 1139.5 p = 0.805
HADS depression	1.7 (2.0)	**3.5 (2.6)**	**5.0 (3.4)**	**U = 1382.0** **p = 0.041**

Vision				
Contrast sensitivity (Pelli Robson) *	1.8 (0.2)	**1.8 (1.2)**	**1.7 (1.1)**	**U = 509** **p < 0.001**
Acuity (LogMar) *	−0.08 (0.2)	−0.09 (0.2)	−0.06 (0.1)	U = 1349 p = 0.074
Colour (D15)	1.3 (1.2)	1.2 (0.9)	1.6 (1.7)	U = 1227 p = 0.088

Cognition				
MOCA	28.8 (1.3)	**28.3 (1.7)**	**27.1 (2.4)**	**U = 763.5** **p = 0.011**
MMSE	29.0 (1.0)	29.0 (1.1)	28.8 (1.4)	U = 942.5 p = 0.208
Mild Cognitive Impairment (MCI)	–	11 (16.4)	15 (45.5)	x^2^ = 8.239 p = 0.004

Disease Specific				
Years from diagnosis	–	3.9 (2.6)	4.7 (2.8)	t = 1.51 p = 0.136
UPDRS total score	–	43.1 (18.7)	49.2 (26.0)	U = 1221 p = 0.399
UPDRS motor score	–	23 (10.1)	23.5 (14.3)	U = 1134 p = 0.837
Hallucinations (within the last week)		9 (13.4)	10 (30.3)	x^2^ = 3.07 p = 0.079
LEDD	–	427.7 (270.5)	491.0 (213.9)	t = 825 p = 0.240
RBDSQ	–	4.4 (2.7)	4.2 (2.2)	U = 1118 p = 0.929
Smell (Sniffin sticks)	–	7.9 (3.2)	6.7 (3.2)	U = 868 p = 0.081

Image Quality metrics				
Coefficient of joint variation^a^	0.7 (0.2)	0.7 (0.2)	0.7 (0.3)	t = -1.14 p = 0.161
Entropy focus criterion^a^	0.7 (0.04)	0.7 (0.03)	0.7 (0.03)	t = -1.01 p = 0.313
Total Signal to noise ratio^b^	7.7 (0.8)	8.2 (1.1)	8.0 (1.0)	t = 1.00 p = 0.317

All data shown are mean (SD) except gender, MCI, and hallucinations. p values reported for the comparison between PD low visual performers and PD high visual performers. In bold significant results. HADS: Hospital anxiety and depression scale; MMSE: Mini-mental state examination; MOCA: Montreal cognitive assessment; UPDRS: Unified Parkinson’s disease rating scale; LEDD: Total Levodopa equivalent dose; RBDSQ: REM sleep behaviour disorder screening questionnaire.

* Best binocular score used; LogMAR: lower score implies better performance, Pelli Robson: higher score implies better performance.

a Higher values imply worse image quality,

b Higher values imply better image quality.

Patients with PD were classified as high visual performers (n = 67) and low visual performers (n = 33), based on performance on two computer-tasks tasks of higher order vision: Cats and Dogs task and Biological motion task. The process of stimulus generation has been previously described in detail (Saygin, 2007, Weil et al., 2019, Weil et al., 2018, Weil et al., 2017); examples of the stimuli are seen in Supplementary Fig. 1. Prior work from our group has shown that performance in these tasks is strongly correlated with dementia risk and worsening cognition at one-year follow up (Leyland et al., 2019, Weil et al., 2019, Weil et al., 2018). Performance in these tasks has significant variability in patients with PD and controls with some overlap in perceptual sensitivity (Weil et al., 2018). For this reason, and to capture patients with consistently poor performance in high-level visual tasks, we classified as low visual performers patients with PD who performed worse than the group median performance on both tasks (n = 33). All other patients with PD were classified as high visual performers (n = 67). Using this classification for visual performance we previously identified widespread white matter micro- and macro-structural abnormalities in PD low visual performers, in the absence of differences in clinically-based dementia risk scores or other cognitive tasks (Zarkali et al., 2020). The present study further extends this work by attempting to clarify the organisational and genetic factors that determine selective vulnerability of specific white matter connections to degeneration.

Participants underwent clinical assessments of motor function, cognition, vision, ophthalmic disease, mood and sleep. Assessment of motor function was performed using the MDS-UPDRS (Goetz et al., 2008). General cognition was assessed using the Mini-Mental State Examination (MMSE) and Montreal Cognitive Assessment (MoCA) (Creavin et al., 2016, Dalrymple-Alford et al., 2010). Mild cognitive impairment (MCI) status in PD participants was assessed using the Litvan second level criteria for MCI in PD (Litvan et al., 2012). Visual acuity was evaluated using the LogMAR (Sloan, 1959). Colour vision was assessed using the D15 (Farnsworth, 1947) and contrast sensitivity using the Pelli-Robson test (Pelli et al., 1988). All participants underwent comprehensive ophthalmic assessment by a consultant ophthalmologist. This included slit-lamp ophthalmic examination and measurement of intra-ocular pressures using Goldman applanation tonometry (Leyland et al., 2019). Sniffin’ Sticks were used to test olfaction (Hummel et al., 1997). Mood was assessed using the Hospital Anxiety and Depression Scale (HADS) (Bjelland et al., 2002) and sleep using the REM Sleep Behaviour Disorder Questionnaire (RBDSQ) (Stiasny-Kolster et al., 2007). Levodopa dose equivalence scores (LEDD) were calculated for PD participants (Tomlinson et al., 2010).

### 2.2 MRI data acquisition

**Fig. 1:**
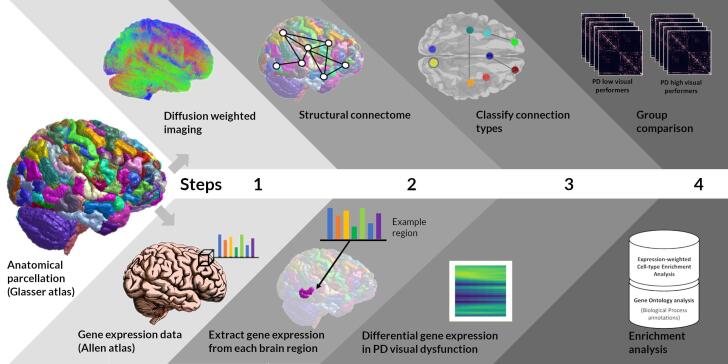
Overview of the study methodology. A1. Anatomically constrained tractography was used to determine white matter streamlines from diffusion weighted imaging (DWI) data for each participant. A2. DWI data were combined with anatomical parcellation of 379 brain regions using the Glasser atlas to generate a connectivity matrix for each participant. A3. Connection types were classified into interhemispheric (between hemispheres), intrahemispheric (within a hemisphere), intramodular (within a cortical module) and subcortial-cortical (between the subcortical regions and a cortical module). A4. Connection strength in different connection types was compared between PD low visual performers and PD high visual performers. B1. Gene expression data were extracted from the Allen Brain atlas and genes passing threshold expression were chosen. B2. Allen atlas samples were mapped into the 180 cortical regions from the left hemisphere according to the anatomical parcellation and an average cortical regional gene expression was calculated for each gene. B3. Partial least squares regression was performed for interhemispheric and basal ganglia-cortical connections in PD low visual performers. The regional gene expression in PD low visual performers associated with interhemispheric and subcortical-cortical white matter loss was calculated based on normalised gene weightings of the second PLS component. B4. Enrichment analysis was performed for the downweighted and upweighted genes that were significantly associated with connection loss for both connection types. Enrichment analysis included Gene Ontology (GO) biological processes, Expression-weighted Cell-type Enrichment analysis (EWCE) and enrichment for specific gene lists for PD, Alzheimer’s, Dementia with Lewy Bodies.

An overview of the study methodology is seen in Fig. 1. All MRI data were acquired on a 3 T Siemens Magnetom Prisma scanner (Siemens) with a 64-channel head coil. Diffusion weighted imaging (DWI) was acquired with the following parameters: b = 50 s/mm2 / 17 directions, b = 300 s/mm2 / 8 directions, b = 1000 s/mm2 / 64 directions, b = 2000 s/mm2 / 64 directions, 2x2x2 mm isotropic voxels, TE = 3260 ms, TR: 58 ms, 72 slices, 2 mm thickness, acceleration factor = 2. Acquisition time for DWI was approximately 10 min. A 3D MPRAGE (magnetization prepared rapid acquisition gradient echo) image (voxel size 1 × 1 × 1 mm, TE: 3.34 ms, TR: 2530 ms, flip angle = 7°) was also obtained and was used to compute intracranial volume using SPM12.

### 2.3 Parcellation

Cortical regions of interest (ROIs) were generated by segmenting a T1- weighted image using the Glasser atlas in FreeSurfer (Glasser et al., 2016). This included 360 cortical regions (180 regions each hemisphere). This atlas was chosen as it is based on a large number of participants (210 healthy adults) which were precisely aligned and was generated by assessing simultaneously four neurobiological properties: architecture, function, connectivity or topography (Glasser et al., 2016). In a recent comparison between different parcellation methods, the Glasser atlas showed good performance across the board compared with other methods and has the advantage of a high spatial resolution (Arslan et al., 2018). 19 subcortical ROIs were generated from the built-in Freesurfer subcortical segmentations (Fischl et al., 2002) resulting to a total of 379 ROIs.

### 2.4 Diffusion weighted imaging pre-processing

Diffusion weighted images underwent denoising (Veraart et al., 2016), removal of Gibbs ringing artefacts (Kellner et al., 2016), eddy-current and motion correction (Anderson, 2006) and bias field correction (Tustison et al., 2010). Diffusion tensor metrics were calculated and constrained spherical deconvolution (CSD) performed, as implemented in MRtrix (Hollander et al., 2016). FreeSurfer Glasser atlas (Glasser et al., 2016) ROIs were warped into diffusion space by registering the T1-weighted image to the diffusion weighted image using FLIRT (Greve and Fischl, 2009). Anatomically constrained tractography was then performed with 10 million streamlines (Smith et al., 2012) using the iFOD2 algorithm with the -backtrack option which allows tracks to be truncated and re-tracked in case of poor termination and the -crop_at_gmwmi option which crops streamline endpoints as they cross the grey matter – white matter interface. Application of the spherical deconvolution informed filtering of tractograms (SIFT2) algorithm (Smith et al., 2015a) with dynamic seeding was then performed to reduce biases. SIFT2 utilises information from the fibre orientation distribution to determine a cross sectional area for each streamline and generate streamline volume estimates between regions whilst utilising the whole connectome (Smith et al., 2015a). The resulting set of streamlines was used to construct the structural brain network.

### 2.5 Structural connectome construction

For each participant, a structural map was generated by determining, for each pair of ROIs, whether they are connected by a streamline from the total reconstructed fibre streamlines; each individual streamline is then weighted by a cross-sectional area multiplier as implemented in SIFT2 (Smith et al., 2015a). The cross-sectional area multiplier for each streamline is calculated in SIFT2 so that the aggregated fibre volumes of all streamlines and their corresponding weights matches the one directly obtained from the diffusion signal throughout the white matter (Smith et al., 2015a). Connections were combined into 379 × 379 undirected and weighted connectivity matrices. We did not apply a threshold on our structural graphs as there is no consensus on the optimal threshold and the chosen threshold can significantly influence results (Garrison et al., 2015, Qi et al., 2015); this is in line with the recommendation by the creators of SIFT2 who advise against the use of matrix thresholding which introduces an arbitrary threshold (Smith et al., 2015a).

### 2.6 Classifying WM connection types

Cortical ROIs of the group-averaged connectivity matrix were partitioned into modules (non-overlapping groups of highly connected nodes) using the data-driven community Louvain algorithm (Blondel et al., 2008) in Brain Connectivity Toolbox (Bullmore and Sporns, 2009), 1,000 permutations and default resolution parameter (γ) of 1.0, resulting in module partition number of 8 (Fig. 1A).

We classified connections as subcortical-cortical: sum of connections between subcortical structures (thalamus, caudate, putamen, pallidum, nucleus accumbens, ventral diencephalon) and ipsilateral cortical modules; interhemispheric: between left and right cortical modules; intrahemispheric: between cortical modules within each hemisphere separately; and, intramodular: within each module. This classification is based on an unbiased, and basic concept of the key connection types. Between, within hemispheres and subcortical-cortical connections have different cytoarchitecture and myeloarchitecture and project to different cortical layers making this a biologically meaningful classification (McColgan et al., 2020, Molyneaux et al., 2007). Connections were normalised to controls using Z-score (within connection regional connectivity was normally distributed across subject; Shapiro Wilk test used to assess normality) and tanh transformed to give a positive WM loss measure, where higher scores represent greater connection loss.

We calculated average connection length in controls, defined as the average streamline length for every pair of brain regions and replicated using topological distance.

### 2.7 Estimating regional gene expression

To correlate regional expression data with connectivity strength, we calculated a connection strength score for each brain region for each connection type. This was defined as the sum of connection weights from this region to subcortical regions (subcortical-cortical score), to regions in the opposite hemisphere (interhemispheric) or regions within the hemisphere (intrahemispheric). These were normalised, tanh-transformed and an average was calculated across PD low visual performers resulting in a single score for each connection type for each region.

We used the Allen Institute for Brain Science (AIBS) transcriptome atlas to extract gene expression data (Hawrylycz et al., 2015). Data from six donors are available for the left hemisphere but only from two donors for the right hemisphere. To achieve good spatial coverage and limit the effect of interindividual differences, we assessed gene expression from left hemisphere samples only (180 regions) (Arnatkevic Iūtė et al., 2019). Each sample was assigned to a region and expression levels for each gene per region were compiled into a 180 × 15745 transcription matrix (Arnatkevic Iūtė et al., 2019); the expression data as well as details on pre-processing can be found https://figshare.com/articles/AHBAdata/6852911.

### 2.8 Statistical analysis

Group differences in demographics were examined using ANOVA for normally distributed variables and Kruskall-Wallis tests for non-normally distributed ones (statistical significance p < 0.05), with post-hoc *t*-test and Mann-Whitney tests respectively.

Group comparison of connection strength was performed using a linear mixed model: age and gender included as covariates, comparisons of interest included PD versus controls and PD low visual performers versus PD high visual performers, false discovery rate (FDR) correction for multiple comparisons (44 connections: 8 subcortical-cortical, 16 interhemispheric, 12 intrahemispheric, 8 intramodular), significance threshold p < 0.05. Additional comparisons were performed to assess effects of general cognitive measures (MOCA, MMSE), and two clinical risk scores for dementia (Liu et al., 2017, Schrag et al., 2017).

Relationship between connection length and WM loss score was investigated using Spearman rank correlation, as both connection length and WM loss score were non normally distributed (normality assessed by Shapiro-Wilk test).

For connection types with significantly reduced connectivity we assessed regional gene expression differences. We used Partial Least Squares (PLS) regression to investigate the association between WM loss in PD low visual performers and gene transcriptome, separately for interhemispheric and subcortical-cortical connections (more details in Supplementary Methods 2). The second PLS component (PLS2) explained the greatest amount of variance of WM connectivity strength. The statistical significance of the variance explained by PLS2 was tested by permuting the predictor variables 1,000 times based on sphere-projection-rotations (Alexander-Bloch et al., 2018). We used bootstrapping to estimate the variability of each gene’s PLS2 weight and tested the null hypothesis of zero weight for each gene (false discovery rate q = 0.05 against 1000 permutations based on sphere-projection-rotations of the WM loss score cortical map). Genes surviving FDR-correction were ranked according to PLS2 weighting and were included in subsequent enrichment analyses; this thresholding was imposed to limit false positive rate in enrichment analyses by only testing for enrichment those genes that had a more positive or more negative weighting than that expected by chance, in keeping with prior studies (Altmann et al., 0000, Morgan et al., 2019, Romero-Garcia et al., 2020). Genes with a negative weighting (downweighted, reflecting a lower expression in regions of reduced structural connectivity) and positive weighting (upweighted, reflecting a higher expression in regions of reduced structural connectivity) were ranked and assessed separately.

### 2.9 Enrichment analysis

Gene ontology (GO) enrichment analysis was performed using g:Profiler (Raudvere et al., 2019), significance threshold corrected p < 0.05, using the g:SCS method for multiple comparison correction which takes into account the set structure underlying gene sets annotated to terms of each organism (for example the overlap between gene ontology terms), providing a tighter threshold to significant results (Raudvere et al., 2019). Genes with a negative weighting (downweighted) and positive weighting (upweighted) were ranked and assessed separately for interhemispheric and subcortical-cortical connections. The reduce and visualize gene ontology tool REViGO was used to visualise significant GO terms (Supek et al., 2011).

To determine whether downweighted or upweighted genes associated with WM loss have higher expression of particular cell types we performed expression-weighted cell-type enrichment analysis (EWCE) (Skene and Grant, 2016). EWCE was performed with target lists comprising the top 20% of the downweighted and upweighted genes (according to their weighting on the second PLS component) for subcortical-cortical, and interhemispheric connection types. Each was run with 100,000 bootstrap lists, controlling for transcript length and GC content, using only major cell-type classes (e.g. “astrocyte”, “microglia”, etc) and using the Benjamini-Hochberg method for correction for multiple comparisons. Single-cell transcription data was derived from AIBS (Hawrylycz et al., 2015) and validated on another dataset (Habib et al., 2017).

We assessed upweighted or downweighted genes for enrichment in specific gene lists using a hypergeometric overlap test. Gene lists that were assessed included: genes associated with a higher risk for PD, from a recent genome wide *meta*-analysis by Nalls et al. (Nalls et al., 2019) using a combination of both nearest genes and quantitative trait locus (QTL) nominated genes from the single nucleotide polymorphism (SNP) data. Genes associated with higher risk of Alzheimer’s disease and genes associated with Dementia with Lewy bodies were similarly chosen from recent genome wide studies (Jansen et al., 2019, Rongve et al., 2019).

### 2.10 Cortical regional enrichment

To assess which cortical regions were enriched for genes in PLS2, we used the ROI weights from the PLS analysis for each connection type.

### 2.11 Robustness analyses

To ensure robustness of our results we performed several replication analyses:

• To ensure that the module count did not influence our results we repeated our analyses using 6 modules (γ = 0.8) and 10 modules (γ = 1.3) as well as a random partition. The random partition was obtained by generated a random, undirected, weighted network with the same number of nodes and edges as our group averaged connectome using the Brain Connectivity Toolbox (Bullmore and Sporns, 2009). We then applied the community Louvain algorithm to the random network with the same parameters as described above. We used the resulting module allocations from the random module for module assignment in each participant’s connectivity matrix.
• We replicated our analysis assessing the effect of length on white matter connectivity loss by assessing topological distance (shortest weighted path length), which takes into account not only distance but also the strength of each individual connection (Supplementary Results 3).
• Recently, it has been suggested that conventional enrichment analyses of brain-wide transcriptomic data may lead to false positive bias for several GO terms due to gene-gene co-expression and spatial autocorrelation in regional neuroimaging data(Fulcher, 2020). To account for this, we only included significantly up- or down-weighted genes compared to 1000 spatially correlated sphere-rotations permutations in our enrichment analyses. Additionally we performed GO enrichment analyses for one random and one spatial-spin permutation of our WM loss score data for both interhemispheric and subcortical-cortical connections and compared the generated GO terms to those of our main analyses.
• As EWCE does not take into account gene ranking (unlike GO enrichment analysis) we wished to include only the most significantly upweighted and downweighted genes in this analysis to ensure that the cell types most responsible are identified; therefore, a gene ranking cut-off was required. The chosen cutoff of top 20% was chosen as it allows for a sufficient number of genes (>50) to be included for both upweighted and downweighted genes for both connection types whilst ensuring that only those genes most significantly associated with white matter connectivity loss are included. We repeated all EWCE analyses using different cut-offs (top 10%, 30% and 50%). In addition we validated all our EWCE findings in two separate, human-derived datasets (Habib et al., 2017, Hawrylycz et al., 2015).

### 2.12 Data availability statement

The connectome and gene expression matrices, along with code to carry out the analyses can be found at https://github.com/AngelikaZa/ConnectomeLength. All data and statistics generated from this study are presented in the manuscript and Supplementary Material. All methods used open source software, all links are included in Supplementary Material.

## 3 Results

134 participants were included: 100 patients with PD (33 low visual performers; 67 high visual performers) and 34 controls. Importantly, PD low and high visual performers did not significantly differ in scan quality (Supplementary Results 1), intracranial volume, disease duration, motor severity or levodopa equivalent dose (Table 1). Mean connectome density was 59.8 (SD = 0.09); this did not significantly differ between PD low visual performers, PD high visual performers and controls (r^2^ = 0.058, p = 0.501).

### 3.1 PD low visual performers show weaker connection strength preferentially in interhemispheric connections

**Fig. 2:**
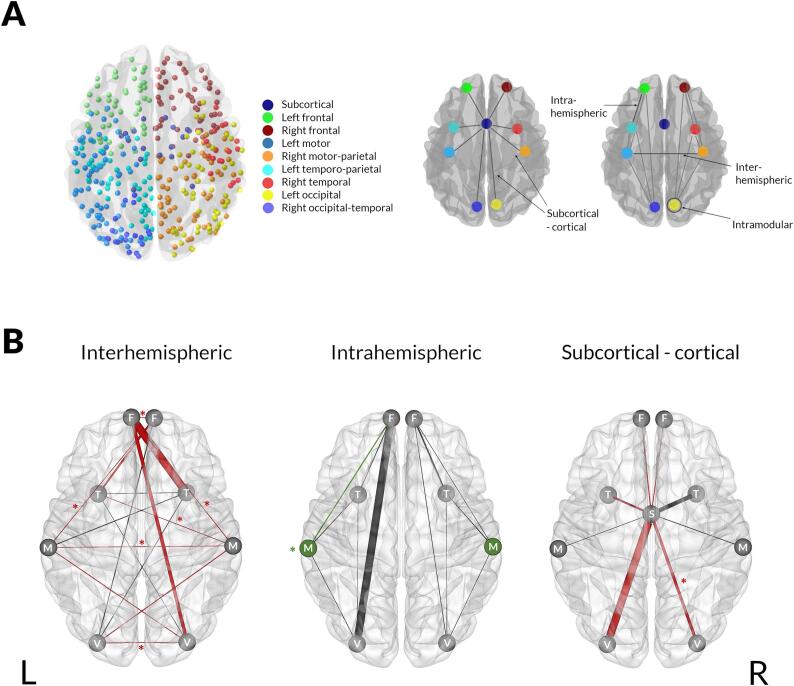
Module assignment and connection types A. Left: Module assignment derived using the Louvain community detection algorithm on the group average control network. This resulted in 8 cortical modules: frontal, left motor, right motor-parietal, left temporal-parietal, right temporal, left occipital and right occipital-temporal. Right: Connections were divided into *Interhemispheric*: defined as the sum of streamline weights (connection strength) between modules in different hemispheres, *Intrahemispheric:* sum of streamline weights (connection strength) between modules in the same hemisphere, *Intramodular*: sum of streamline weights (connection strength) within the same cortical module, and *Subcortical-cortical*: the sum of streamline weights (connection strength) from subcortical regions to a cortical module. B. Hierarchy of connection vulnerability. Mixed linear model results for connectome analysis: patients with Parkinson’s (PD) low visual performers vs. PD high visual performers. Interhemispheric connections are most affected, followed by subcortical-cortical connections, with intrahemispheric and intramodular connections showing preserved connectivity strength. Figure illustrates the individual connections showing changes in connectivity strength in PD low visual performers. The thickness of the line represents absolute effect size (difference in connectivity strength in PD low visual performers). Red: Reduced connectivity strength, Green: Increased connectivity strength, Grey: No significant difference in conncectivity strength. F: frontal, T: temporal; M: motor-parietal; V: occipital, B: Subcortical. (For interpretation of the references to color in this figure legend, the reader is referred to the web version of this article.)

First, we examined how different connection subtypes differed between groups. Between PD and control participants, there were no significant differences (FDR-corrected p-value: q < 0.05). In PD low visual performers, connection strength was significantly weaker for interhemispheric connections (6/16 connections, 37.5%) compared to PD high visual performers. These included connections between the two occipital modules, two frontal modules and two motor/parietal modules, and connections from each frontal to contralateral motor modules (Fig. 2B, Table 2). Connection strength in subcortical-cortical connections was also weaker in PD low visual performers compared to PD high visual performers (1/8 connections 12.5%), with the connection between right subcortical and occipital/temporal module surviving FDR-correction (Fig. 2B, Table 2).

**Table 2:** Differences in connection strength in patients with Parkinson’s disease and low visual performance for different connection types.

**Interhemispheric connections**	**T**	**β**	**95% CI**	**p value**	**q value**
Left occipital to right occipital-temporal	**−2.837**	**−260.9**	**−441.1, −80.7**	**0.005**	**0.038**
Left occipital to right motor-parietal	−2.074	−811.5	−1578.5, −44.6	0.038	0.095
Left occipital to right temporal	−1.779	−6026.4	−12666.9, 614.1	0.075	0.150
Left occipital to right frontal	−1.209	−13397.5	−35111.2, 8316.1	0.227	0.322
Left temporoparietal to right occipital-temporal	−1.733	−357.3	−761.5, 46.8	0.083	0.159
Left temporoparietal to right motor-parietal	**−2.746**	**−313.3**	**−436.9, −89.7**	**0.006**	**0.038**
Left temporoparietal to right temporal	−0.687	−408.2	−1572.8, 756.4	0.492	0.610
Left temporoparietal to right frontal	−2.287	−2607.4	−4842.2, −372.6	0.022	0.065
Left motor to right occipital-temporal	−2.19	−596.8	−1130.8, −62.8	0.029	0.080
Left motor to right motor-parietal	**−3.569**	**−108.3**	**−167.8, −48.8**	**0**	**0.000**
Left motor to right temporal	−1.404	−788.8	−1890.4, 312.7	0.16	0.282
Left motor to right frontal	**−2.72**	**−415.1**	**−714.2, −116.0**	**0.007**	**0.038**
Left frontal to right occipital-temporal	−2.398	−5578	−10137.8, −1018.3	0.016	0.058
Left frontal to right motor-parietal	**−3.048**	**−482.7**	**−793.2, −172.3**	**0.002**	**0.022**
Left frontal to right temporal	−2.378	−13754.1	−25090.6, 2417.5	0.017	0.058
Left frontal to right frontal	**−3.224**	**−92.881**	**−149.4, −36.4**	**0.001**	**0.015**
**Subcortical-cortical connections**	**t**	**β**	**95% CI**	**p value**	**q value**
Left subcortical to occipital	−2.339	−330.1	−606.8, −53.4	0.019	0.060
Left subcortical to temporoparietal	−2.447	−92.5	−166.6, −18.4	0.014	0.058
Left subcortical to motor	−1.284	−28.1	−70.9, 14.8	0.199	0.302
Left subcortical to frontal	−2.058	−46.7	−91.2, −2.2	0.04	0.095
Right subcortical to occipital-temporal	**−2.731**	**−128.5**	**−220.6, 36.3**	**0.006**	**0.038**
Right subcortical to motor-parietal	−1.857	−36.7	−75.5, 2.0	0.063	0.132
Right subcortical to temporal	−1.298	−178.3	−447.6, 90.9	0.194	0.302
Right subcortical to frontal	−2.042	−42.2	−82.7, −1.7	0.041	0.095
**Intrahemispheric connections**	**t**	**β**	**95% CI**	**p value**	**q value**
Left occipital to temporoparietal	−0.334	−14.1	−96.9, 68.7	0.738	0.833
Left occipital to motor	0.218	25.5	−203.7, 254.7	0.827	0.859
Left occipital to frontal	−1.882	−1383	−2823.5, 57.5	0.06	0.132
Left temporoparietal to motor	1.361	50.6	–22.3, 123.4	0.174	0.294
Left temporoparietal to frontal	−0.943	−68.5	−210.9, 73.9	0.346	0.461
Left motor to frontal	2.473	110.2	22.8, 197.6	0.013	0.058
Right occipital-temporal to motor-parietal	0.264	10.1	−65.1, 85.4	0.792	0.859
Right occipital-temporal to temporal	−0.203	−15.2	−161.7, 131.3	0.839	0.859
Right occipital-temporal to frontal	−0.451	−32.1	−230.4, 144.2	0.652	0.755
Right motor-parietal to temporal	−0.896	−71.9	−229.1, 85.4	0.37	0.479
Right motor-parietal to frontal	1.313	46.5	−22.9, 115.9	0.189	0.302
Right temporal to frontal	−0.676	−182.8	−713.2, 347.5	0.499	0.610
**Intramodular connections**	**t**	**β**	**95% CI**	**p value**	**q value**
Left occipital	0.245	1	−7.2, 9.3	0.806	0.859
Left temporoparietal	1.58	5.3	−1.3, 11.9	0.114	0.209
Left motor	**3.3**	**17.1**	**6.9, 27.2**	**0.001**	**0.015**
Left frontal	0.944	5.4	−5.8, 16.6	0.345	0.461
Right occipital-temporal	−0.005	−0.1	−4.9, 4.9	0.996	0.996
Right motor-parietal	2.383	11.9	2.1, 21.8	0.017	0.058
Right temporal	0.641	7.6	−15.6, 30.8	0.522	0.621
Right frontal	1.231	6.8	−4.0, 17.5	0.218	0.320

t: t value for the specific intercept on the mixed linear model (covariates: age and gender); q value: FDR corrected p value;

* negative value indicates lower connection strength in PD low visual performance compared with PD high visual performance. In bold values that are statistically significant (corrected for multiple comparisons)

In contrast, no significant group differences were seen in intrahemispheric connections and only one intramodular connection differed in PD low visual performers compared to PD high visual performers (8.3%): higher connection strength within the left motor module (Fig. 2B, Table 2). This observed preferential pattern of connectivity loss for interhemispheric and, to a lesser extent, subcortical-cortical connections was preserved using different module counts (Supplementary Results 2); in contrast, we saw no significant group differences on replication analysis with random module allocations.

General measures of cognition (MMSE and MOCA) as well as two clinically-derived scores for dementia risk (Liu et al., 2017, Schrag et al., 2017) were not significantly correlated (after correction for multiple comparisons) with structural connectivity differences in any connection types.

However, across participants with PD, a composite interhemispheric WM loss score (the sum of all WM loss scores across all interhemispheric connections, that showed the most significant differences in PD low visual performers versus PD high visual performers) was significantly correlated with MOCA performance (rho = 0.213, p = 0.034) but not MMSE (rho = 0.184, p = 0.067).

### 3.2 Loss of connection strength in PD low visual performers is weakly associated with connection length in health

To investigate whether connection length was a driver for selective vulnerability, we calculated average connection length of the four connection subtypes in controls. Length weakly but significantly differed across connection types (df(3, 88000), r^2^ = 0.066, p < 0.001): subcortical-cortical connections were longest, followed by interhemispheric, intrahemispheric and finally intramodular connections (distribution of length per connection type in Supplementary Results 2).

**Fig. 3:**
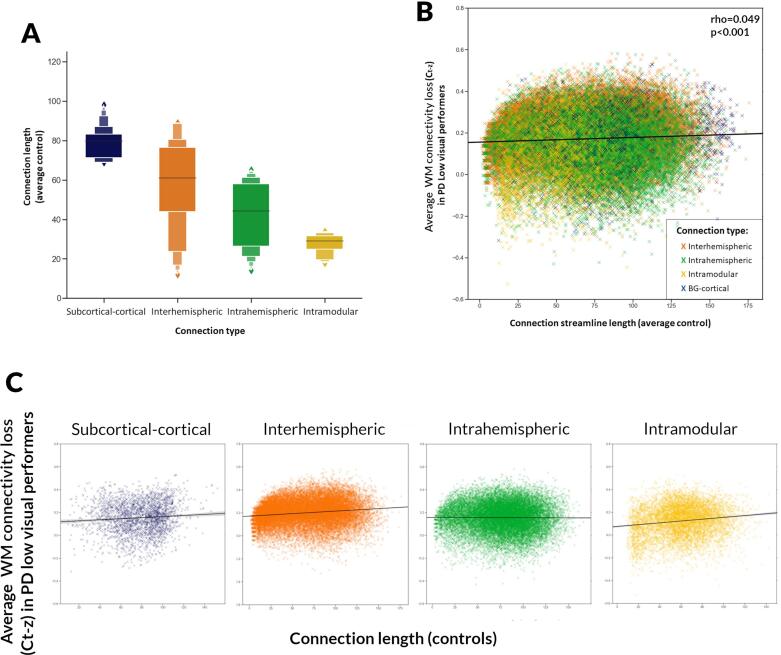
Correlation between connection length and white matter connectivity loss in PD low visual performers. A. Distribution of connection length for different connection types. Average connection length was calculated for each connection in control participants. B. Correlation between connection length and white matter loss in PD low visual performers. Connection strength was normalised against control participants using Z-scores, then transformed into positive connectivity loss measures using a tanh transform. Average transformed connection strength score for PD low visual performers is plotted against connection-weighted path length for average control, and Spearman rank correlations were performed. Connections are color-coded according to type. C. Correlation between connection length and white matter loss in PD low visual performers for individual connection types.

Connection length across all connection types showed a weak significant correlation with WM loss score in PD low visual performers (rho = 0.049, p < 0.001) (Fig. 3B). The correlation between connection length in controls and WM loss scores in PD low visual performers were stronger within specific connection types: subcortical-cortical (rho = 0.122, p < 0.001), interhemispheric (rho = 0.191, p < 0.001), intrahemispheric (rho = 0.015, p = 0.031) and intramodular connections (rho = 0.123, p < 0.001) (Fig. 3C). To ensure that module selection was not influencing results, analyses were replicated using 6 and 10 modules (Supplementary Results 3).

Topological distance also differed across connection types, showing similar correlation with WM loss scores (rho = 0.213, p < 0.001). (Supplementary Results 4).

### 3.3 Divergent regional gene expression patterns are linked with interehemispheric and subcortical-cortical connectivity loss in PD with low visual performance

Given that WM connectivity loss was only seen in interhemispheric and subcortical-cortical connections, we assessed gene expression patterns related to WM loss in PD low visual performers for those connection types. For both, PLS2 explained the largest percentage of variance in WM loss in PD low visual performers (interhemispheric 13.8%, subcortical-cortical 9.5%; both permutation test p < 0.001). Therefore, we used PLS2 scores to rank genes surviving FDR-correction in permutation testing (individual PLS2 weightings of each gene in Supplementary Table S3).

**Fig. 4:**
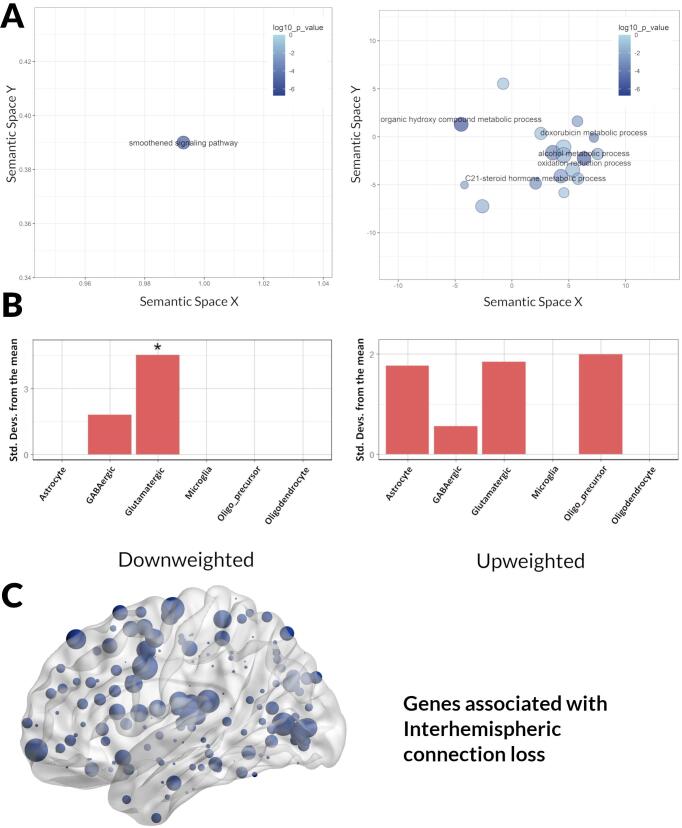
Enrichment analyses results for down- and up-weighted genes associated with interhemispheric connection loss in PD low visual performers. A. Significant gene ontology (GO) terms for biological processes associated with the second component of the partial least squares analysis plotted in semantic space, where similar terms are clustered together (Left panel: downweighted genes; Right panel: upweighted genes). The top five most significant GO terms are labelled for each analysis. Redundant GO terms have been excluded. Markers are scaled based on the log10 q value for the significance of each GO term. Larger and darker circles are highly significant, while smaller and lighter circles are less significant (see colour bar). B. Expression-weighted cell-type enrichment analysis (EWCE) using the AIBS dataset. EWCE uses single cell transcriptomes to generate the probability distribution associated with a gene list having an average level of expression within a cell type. Data are presented as standard deviations from the mean. Standard deviations from the mean indicate the distance of the mean expression of the target gene lists from the mean expression of the bootstrap replicates. Marked with * are statistically significant (FDR corrected) results. C. Region of interest weights for partial least squares regression analyses. Brain regions displayed on brain mesh. Size of region indicates size of region of interest weight. Figure plotted using BrainNet Viewer [45].

**Fig. 5:**
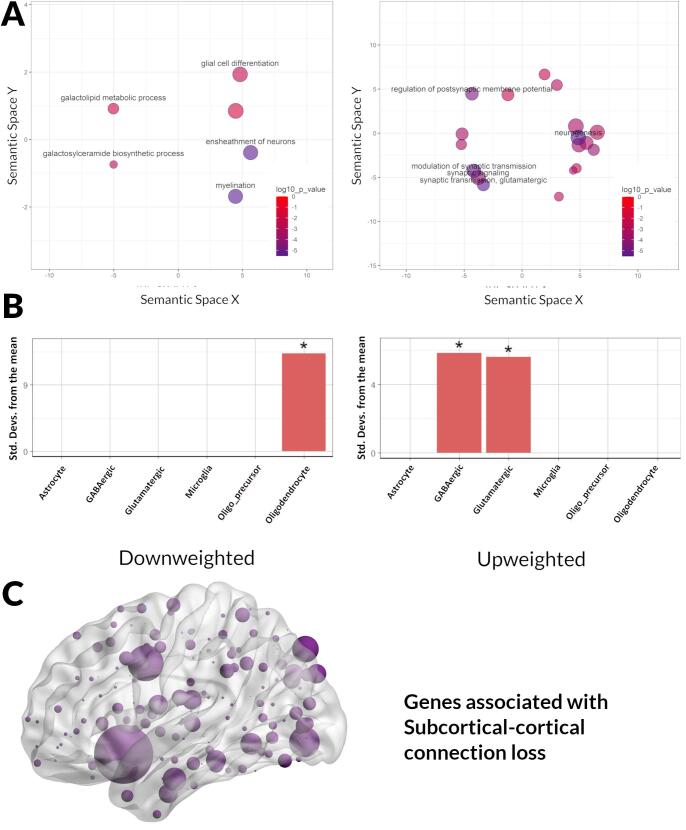
Enrichment analyses results for down- and up-weighted genes associated with subcortical-cortical connection loss in PD low visual performers. A. Significant gene ontology (GO) terms for biological processes associated with the second component of the partial least squares analysis plotted in semantic space, where similar terms are clustered together (Left panel: downweighted genes; Right panel: upweighted genes). The top five most significant GO terms are labelled for each analysis. Redundant GO terms have been excluded. Markers are scaled based on the log10 q value for the significance of each GO term. Larger and darker circles are highly significant, while smaller and lighter circles are less significant (see colour bar). B. Expression-weighted cell-type enrichment analysis (EWCE) using the AIBS dataset. EWCE uses single cell transcriptomes to generate the probability distribution associated with a gene list having an average level of expression within a cell type. Data are presented as standard deviations from the mean. Standard deviations from the mean indicate the distance of the mean expression of the target gene lists from the mean expression of the bootstrap replicates. Marked with * are statistically significant (FDR corrected) results. C. Region of interest weights for partial least squares regression analyses. Brain regions displayed on brain mesh. Size of region indicates size of region of interest weight. Figure plotted using BrainNet Viewer [45].

We saw different GO term enrichment patterns for different connection types. Interhemispheric connections downweighted genes were enriched for the term smoothened signalling pathway (Fig. 4A), whilst for upweighted genes the most significant terms included organic hydroxy metabolic process, oxidation reduction process and alcohol metabolic process (Fig. 4A). (Table 3 for five most enriched terms (full list of enriched GO terms in Supplementary Table S3). For subcortical-cortical connections the most significant GO term enrichments amongst downweighted genes included myelination, ensheathment of neurons, glial-cell differentiation and galactosylcelcarmide and galactolipid metabolic process (Fig. 5A). Upweighted genes in subcortical-cortical connections were enriched for terms including synaptic signalling, modulation of synaptic transmission, regulation of postsynaptic membrane potential, neurogenesis, and glutamatergic synaptic transmission (Fig. 5A).

**Table 3:** Gene Ontology (GO) terms for biological processes associated with significantly Downweighted and Upweighted genes from the second component of partial least squares regression (PLS2).

Interhemispheric connections					
Downweighted Genes					
GO term	Description	q value	B	N	b
GO:0007224	smoothened signaling pathway	1.89E-02	146	734	19
Upweighted Genes					
GO term	Description	q value	B	N	b
GO:1901615	organic hydroxy compound metabolic process	1.87E-07	557	239	30
GO:0006066	alcohol metabolic process	1.67E-06	377	192	21
GO:1902644	tertiary alcohol metabolic process	9.84E-06	20	19	4
GO:1901617	organic hydroxy compound biosynthetic process	3.14E-05	268	134	14
GO:0006695	cholesterol biosynthetic process	7.50E-05	71	125	8
Subcortical-cortical connections					
Downweighted Genes					
GO term	Description	q value	B	N	b
GO:0042552	myelination	2.90E-06	140	146	12
GO:0007272	ensheathment of neurons	3.70E-06	143	146	12
GO:0008366	axon ensheathment	3.70E-06	143	146	12
GO:0019375	galactolipid biosynthetic process	2.60E-03	6	76	3
GO:0006682	galactosylceramide biosynthetic process	2.60E-03	6	76	3
Upweighted Genes					
GO term	Description	q value	B	N	b
GO:0035249	synaptic transmission, glutamatergic	2.07E-04	105	762	19
GO:0022008	neurogenesis	2.31E-04	1683	1378	187
GO:0050804	modulation of chemical synaptic transmission	3.77E-04	457	762	45
GO:0099177	regulation of *trans*-synaptic signaling	4.01E-04	458	762	45
GO:0060078	regulation of postsynaptic membrane potential	8.56E-04	149	762	22

The top five most significant GO terms are displayed for each connection type. Full GO terms are presented in Supplementary Table S5.

q value: log10 of the FDR adjusted p value; B: Total number of genes associated with a specific GO term;

b:Number of genes in the intersection ; N: Number of genes in the target set (query size).

In contrast, results from enrichment analyses on a random and spatial-spin null model for interhemispheric and subcortical-cortical connections showed divergent results (See Supplementary Results 5 for details). For interhemispheric connections, downweighted genes of the spatial-spin null model were most enriched in terms related to synapse organisation and regulation and upweighted genes were most enriched in terms such as protein targeting and mitochondrion organisation; both fully divergent from our main analyses. For subcortical-cortical connections, the most enriched terms for downweighted genes of the spatial-spin null model were electron transport chain, oxidation–reduction process and small molecule metabolism, whilst upweighted genes were enriched in terms related to RNA metabolism and biosynthesis, again qualitatively different to our main results. Details on the most enriched terms of the null models can be seen in Supplementary Results 5 and the full GO terms in Supplementary Table S6.

### 3.4 Spatial variation of gene expression profile for loss of interhemispheric and subcortical connections

We next explored the spatial pattern of each gene expression profile in the brain. We analysed each ROI PLS2 weights for both connection types; higher ROI weights reflect greater gene enrichment (Supplementary Table S4 for raw ROI weights). For interhemispheric connections, regions with highest weights were predominantly in occipital, frontal, and parietal cortices (Fig. 4C). In contrast, for subcortical-cortical connections, regions with highest weights were predominantly in frontal, motor, and occipital cortices (Fig. 5C).

### 3.5 Cell types and risk variants linked with WM connectivity loss in PD low visual performers

We investigated whether the most upweighted and downweighted genes associated with interhemispheric and subcortical-cortical connection loss were enriched in specific cell types. Cell-type expression profiles were defined using human-derived single-nucleus data from AIBS and enrichment determined using EWCE. For interhemispheric connections, we found that the top 20% most downweighted genes (1 5 8) enriched in glutamatergic neurons whilst the top 20% most upweighted genes (1 5 8) were not significantly enriched in any cells (Fig. 4B). For subcortical-cortical connections, we found that the top 20% most downweighted genes (153 genes) enriched in oligodendrocytes while the top 20% most upweighted genes (2 9 3) enriched in glutamatergic and GABAergic neurons (Fig. 5B).These enrichment patterns were preserved both when different proportions of the most upweighted and downweighted genes were used (i.e. top 10%, 30% and 50%) and when cell-type profiles were defined using a different human-derived single-nucleus dataset (Supplementary Results 5).

Subsequently, we investigated whether the most upweighted and downweighted genes in interhemispheric and subcortical-cortical connections were enriched for genes linked to increased risk of PD, AD or DLB. Upweighted genes for subcortical-cortical connections showed a trend for enrichment for genes associated with increased PD risk (15 shared genes, p = 0.044 (uncorrected for multiple comparisons), Supplementary Results 6) but not those associated with AD (p = 0.762) or DLB (p = 0.998). The subcortical-cortical downweighted and interhemispheric up- and downweighted genes were not significantly enriched for any risk genes.

## 4 Discussion

Here we demonstrate the hierarchy of WM connection loss accompanying visual dysfunction in PD, an early marker of Parkinson’s dementia, and shed light on the organisational and genetic factors that influence WM vulnerability.

In PD low visual performers, we found a specific pattern of WM connection loss with preferential loss of primarily interhemispheric and to lesser extent, subcortical-cortical connections, but preserved intrahemispheric and intramodular connectivity.

Given that both subcortical-cortical and interhemispheric connections serve topologically distant brain regions, we investigated whether connection length was a significant driver for the selective vulnerability of these connection types. We showed that normal connection length does correlate, albeit weakly, with WM loss in PD low visual performers, with longer connections being more affected. This is in accordance with imaging data showing WM changes within the corpus callosum, posterior thalamic radiations and fronto-occipital fasciculi in PD with visual dysfunction (Zarkali et al., 2020) and pathology data showing vulnerability to PD dementia preferentially involving cells with long axonal projections (Hale and Lowry, 2011, Perry et al., 1985, Wu et al., 2014). However, the weak correlation of connection length to connectivity loss implies that the type rather than the length of connection, may be a more significant driver of connectivity loss.

Different connection types reflect different neuronal populations; interhemispheric connections mostly comprise intratelencephalic neurons, ipsilateral subcortical-cortical connections mostly comprise pyramidal tract neurons whilst short intramodular connections mostly comprise inhibitory interneurons (Harris and Shepherd, 2015, Molyneaux et al., 2007). These differentiate at different times during development, project to different cortical laminar layers and show distinct gene expression profiles and levels of myelination (Anderson et al., 2010, Harris and Shepherd, 2015, Molyneaux et al., 2007).

To investigate the biological processes driving selective regional vulnerability in PD low visual performers, we assessed the cortical gene expression profile of interhemispheric and subcortical-cortical connections. Additionally, given that changes in regional gene expression can reflect changes in cell type proportions between regions, we investigated whether genes associated with WM loss in PD low visual performers are more highly expressed in one cell type than another and found a distinct pattern of cell type-specific expression.

We found that vulnerability to subcortical-cortical connection loss in PD low visual performers was associated with downweighted genes related to myelination and enriched in oligodendrocytes. Oligodendrocyte dysfunction could play a key role in WM loss in PD dementia, with recent evidence suggesting that PD heritability is enriched in oligodendrocytes, in addition to cholinergic and monoaminergic neurons and enteric neurons (Bryois et al., 2020). Another explanation is that subcortical-cortical connections, which have large connection length but small topological distance, are heavily myelinated. Amongst these oligodendrocyte-rich regions, those with lower oligodendrocyte expression and more poorly myelinated in health (for example dopaminergic neurons (Sulzer and Surmeier, 2013)) could be more “at risk” in the presence of neurodegeneration.

Upweighted genes associated with subcortical-cortical connection loss were enriched in terms related to synaptic signalling and chemical synaptic transmission, particularly glutamate; these genes were also enriched in GABAergic and glutamatergic neurons. Alpha-synuclein monomers play a role in synaptic function and neurotransmitter release (Abeliovich et al., 2000, Cabin et al., 2002) with oligomers involved in synaptic dysfunction (Diógenes et al., 2012, Rockenstein et al., 2014) and intrastriatal injections of alpha-synuclein are associated with reduced glutamate receptor activity (Durante et al., 2019). Our findings further support the importance of synaptic dysfunction in PD.

In contrast, interhemispheric connections showed a different gene expression pattern, in keeping with a different neuronal population being affected. Downweighted genes associated with interhemispheric connection loss were enriched in a single GO term, smoothened signalling pathway, and in glutamatergic neurons. The smoothened signalling pathway is crucial for the brain development, particularly that of dopaminergic neurons and dysfunction, and has been previously implicated in Parkinson’s disease (Gonzalez-Reyes et al., 2012). In addition to its role within the nigrostriatal circuit, the smoothened signalling pathway has been recently shown to play a role in plasticity and neuronal regeneration in the adult brain (Petrova and Joyner, 2014, Yao et al., 2016) as well as local glial proteostasis. Indeed, impairment of the smoothened signalling pathway in a *Drosophila* model of Alzheimer’s disease leads to aging and reduced lifespan, while activation of the pathway results in reduced amyloid accumulation (Rallis et al., 2020). In the long distance neurons that form interhemispheric connections, local regulatory processes are particularly important, due to their morphology and size (Holt et al., 2019); lower expression of genes related to the smoothened signalling pathway may contribute to impaired local proteostasis, making them more vulnerable to aberrant protein aggregation and misfolding.

Relatively higher expression, linked to interhemispheric WM loss, of genes involved in metabolic processes, such as organic hydroxy compound metabolism, could reflect the added metabolic burden of maintaining these long-distance connections, making these connections more vulnerable to degeneration. Indeed, highly connected brain regions or “hubs” are characterised by higher expression of metabolic and mitochondrial genes (Fulcher and Fornito, 2016, Vértes et al., 2016).

Recent studies have implicated the role of the innate immune system, specifically microglia disfunction, in PD (Ouchi et al., 2005, Yun et al., 2018). We did not find enrichment for microglia-specific genes associated with structural connectivity loss in our cohort, similar to recent PD heritability studies (Bryois et al., 2020). This suggests that factors other than background microglia expression, for example aberrant activation, drive degenerative losses of white matter in PD.

Our findings are likely to be disease-specific. We showed relative enrichment of upweighted genes related to subcortical-cortical connection loss for PD but not AD risk genes, where different pathological mechanisms are likely to drive WM loss. Although we did not find enrichment for DLB risk genes (where mechanisms may be linked), that gene list was derived from a small GWAS study (Rongve et al., 2019). Studies correlating imaging metrics with regional gene expression for other diseases show different results implying disease-specificity. For example, in schizophrenia, WM loss is related to genes involved in cell-to-cell signalling (Romme et al., 2017). In contrast, in Huntington’s disease, a form of neurodegeneration with a more similar phenotype of subcortical dementia to Parkinson’s disease, gene expression patterns associated with WM loss are more similar to those we found in PD low visual performers (McColgan et al., 2018).

## 5 Limitations

Gene expression data from the Allen atlas were derived from healthy donors without neuropsychiatric disease, and may differ in PD. Postmortem data suggests that patients with PD show different gene expression within the substantia nigra compared to controls (Mariani et al., 2016, Simunovic et al., 2009); we used cortical gene expression data to mitigate for this. Moreover, our main comparison of interest is between PD with visual dysfunction and PD with preserved vision. Due to data availability in the Allen atlas, we used only left hemisphere data. There is no reason to expect cortical differences in gene expression between hemispheres, but this could be examined in future work. We could not validate our gene expression findings using other transcriptome datasets as the two other available human transcriptome atlases (UK Brain Expression Consortium (Ramasamy et al., 2014) and Human Brain Transcriptome project (Kang et al., 2011)) were derived from only small number of cortical regions.

Diffusion tractography is a relatively indirect marker of WM connectivity. However, diffusion imaging is currently the only available technique to assess structural connectivity in vivo. We used constrained spherical deconvolution, which is more reliable in assessing crossing fibers (Smith et al., 2012) and the SIFT2 algorithm which is more representative of underlying biology (Smith et al., 2015a, Smith et al., 2015b). Although all imaging (both diffusion weighted and structural) were visually inspected for the presence of structural abnormalities and no significant vascular disease was seen, we did not systematically assess and control for the presence of white matter hyperintensities. Future studies should clarify the effect, if any, of white matter hyperintensities on structural connectivity measures.

In this study, we used a modular approach to simplify the interpretation of large numbers of brain connections, classifying connections as interhemispheric, subcortical-cortical, intrahemispheric and intramodular. This is a biologically meaningful approach (Molyneaux et al., 2007), which was selected based on prior work (Zarkali et al., 2020). However, it is worth noting that summing connections from multiple regions may result in over- or under-estimation of between group differences.

Participants with PD were scanned on their usual dopaminergic medications. It is unlikely that levodopa affects structural connectivity, as fractional anisotropy is not influenced by levodopa (Chung et al., 2017). Finally, our study is cross-sectional. Longitudinal studies in patients with visual deficits who progress to dementia will provide further insights into the temporal order of WM loss and biological processes involved.

## 6 Conclusion

We show that WM loss in PD patients with visual dysfunction, who are at risk of Parkinson’s dementia, exhibits a specific pattern, with interhemispheric connections preferentially affected. In addition, we show that WM loss in PD low visual performers is associated with distinct gene expression patterns, linked with different biological processes and different cell types and invoking high metabolic burden as a driver for involvement for selective regions. These findings elucidate the earliest changes in WM connectivity of PD dementia and shed light onto the underlying pathological processes that may drive them.

## CRediT authorship contribution statement

**Angeliki Zarkali:** Conceptualization, Data curation, Methodology, Software, Investigation, Formal analysis, Writing - original draft, Writing - review & editing, Visualization. **Peter McColgan:** Conceptualization, Methodology, Resources, Writing - review & editing. **Mina Ryten:** Methodology, Resources, Writing - review & editing. **Regina H. Reynolds:** Methodology, Resources, Writing - review & editing. **Louise-Ann Leyland:** Project administration, Data curation, Writing - review & editing. **Andrew J. Lees:** Conceptualization, Methodology, Writing - review & editing. **Geraint Rees:** Conceptualization, Methodology, Writing - review & editing. **Rimona S. Weil:** Conceptualization, Methodology, Funding acquisition, Project administration, Supervision, Writing - review & editing.

## Declaration of Competing Interest

The authors declare that they have no known competing financial interests or personal relationships that could have appeared to influence the work reported in this paper.
