# Supplementary material for: Dementia risk in Parkinson’s disease is associated with interhemispheric connectivity loss and determined by regional gene expression
Source: Neuroimage Clin. 2020 Oct 15;28:102470. doi: 10.1016/j.nicl.2020.102470 (PMC7581968; doi:10.1016/j.nicl.2020.102470)
Supplement: Supplementary data 1 [file mmc1.pdf]

Supplementary Material

Dementia risk in Parkinson's is associated with interhemispheric connectivity loss and determined by regional gene expression

Angeliki Zarkali MBBS[1], Peter McColgan PhD[2], Mina Ryten PhD[3], Regina Reynolds MSc[3], Louise-Ann Leyland PhD[1], Andrew J. Lees PhD[4], Geraint Rees PhD[5,6], Rimona S. Weil PhD[1,6,7]

Contents

Supplementary Methods 2

1. Computer-based visual tasks 2

2. Connection length and topological distance in healthy controls 3

3. Partial Least Regression 3

Supplementary Results 4

1. Motion and scan quality **4**

2. Connection length in healthy controls **5**

3. Replication analysis: Different module counts **5**

4. Replication analysis: Topological Distance **8**

5. Replication analysis: Enrichment analysis of random and spatial-spin nulls **11**

6. Replication analysis: EWCE with different single cell transcription dataset **13**

6. Table S1. Genes with common variations resulting to increased risk for Parkinson's disease overlapping with upweighted genes for subcortical-cortical connections **18**

Additional Files 18

URLs: 18

References 19

Supplementary Methods

1. Computer-based visual tasks

Figure S1. **Examples of the visual stimuli used**
A: Cats and Dogs task: Example stimulus
B: Biological Motion task: Example frame from an animation depicting a person walking

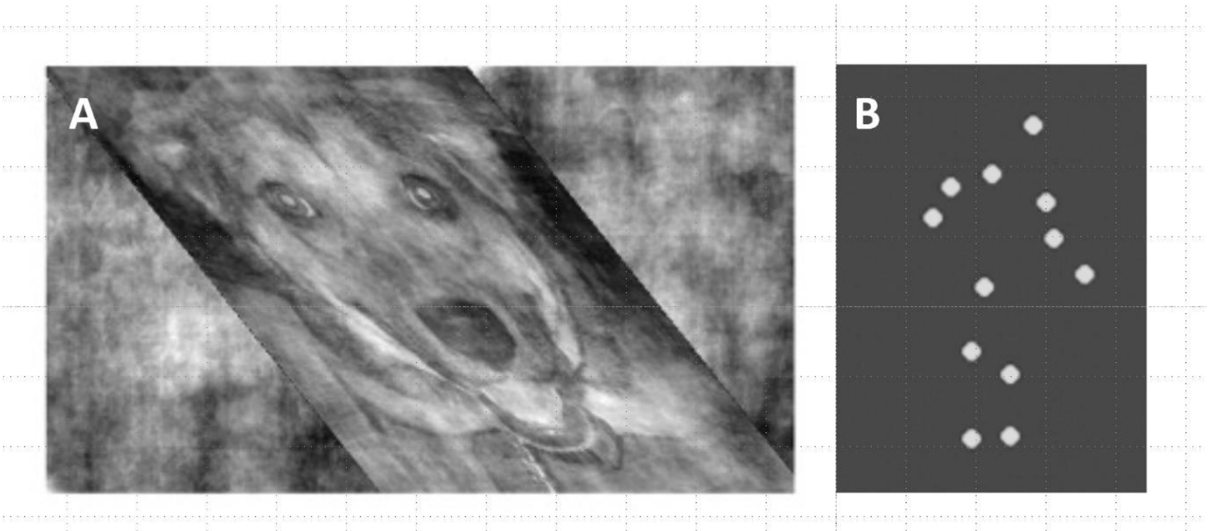

Figure S2. **Performance of patients with Parkinson's disease in the visual tasks**.
A. Distribution of performance in the Cats and Dogs task. Orange line highlights the median performance in this task. Performance was not significantly different between PD and controls (rho=0.168, p=0.057).
B. Distribution of performance in the Biological motion task. Orange line highlights the median performance in this task. Performance was not significantly different between PD and controls (rho=0.025, p=0.778).
C. Distribution of the combined Z score of performance in the Biological motion and Cats and Dogs task ($Z_{score} = Z_{Biolmotion} + Z_{cats\&dogs}$). The distribution on the left of the red line represents the patients who performed worse than the median (left of the orange lines in A and B) for both the Cats and Dogs and the Biological motion task. These were classified as PD low visual performers.

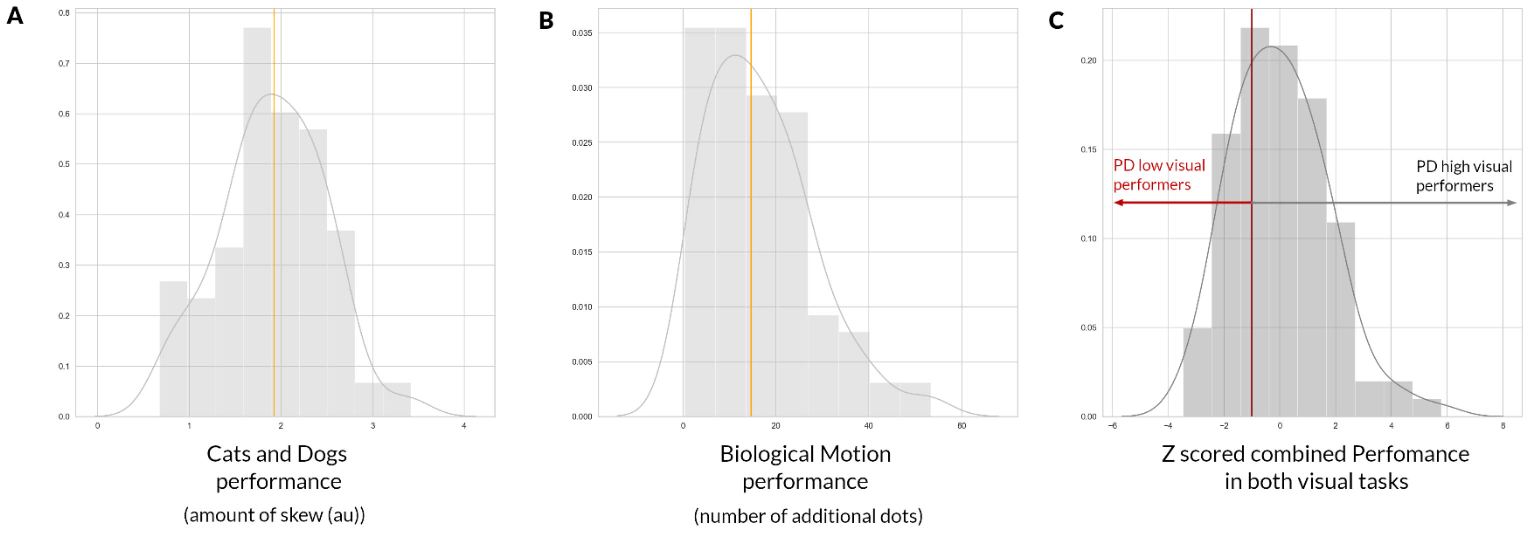

2. Connection length and topological distance in healthy controls

Connection length was defined as the streamline length between two brain regions. This was calculated for each connection for each participant in the individual connectivity matrix and then an average connection length for each connection was derived for all control participants. Topological distance was defined as the shortest weighted path (Figure S6). This was computed for every pair of brain regions in the averaged healthy control brain network using the Brain Connectivity Toolbox (Bullmore and Sporns, 2009). The weighted connectivity matrix was first converted to a connection-length matrix with higher connection weights interpreted as shorter lengths. Dijkstra's algorithm was then used to calculate the shortest weighted path between each pair of brain regions (Dijkstra, 1959).

3. Partial Least Regression

Partial Least Squares (PLS) regression was used to investigate the association between white matter connectivity loss in PD participants with low visual performance and the gene transcriptome of the healthy brain. This was performed in Python 3 using the hoggorm library. PLS regression is a multivariate technique used to identify associations between response and predictor variables. It is particularly useful when predictors are highly colinear such as gene expression data and when the number of predictors exceeds that of the observations (Rosipal and Krämer, 2006). It has also been extensively used in correlating imaging-derived metrics with transcriptomic data (Rubinov *et al.*, 2015; Vértes *et al.*, 2016; Whitaker *et al.*, 2016; McColgan *et al.*, 2018; Morgan *et al.*, 2019; Romero-Garcia *et al.*, 2019). Our predictor variable was a 15745 gene x 180 ROI matrix. Given that we observed reduced connectivity in subcortical-cortical and interhemispheric connections only, we used these connections types (1 x 180 vector for each connection representing white matter connectivity loss for this connection type for each cortical ROI) as response variables. As the greatest amount of variance was explained by the second PLS component (PLS2) across both connection types, genes were ranked based on their contribution to the second component. We performed permutation testing to assess whether PLS2 results explained a significantly higher proportion of variance in white matter atrophy than expected by chance. To do this we reordered the predictor matrix in term of ROIs based on sphere-rotations (Alexander-Bloch *et al.*, 2018) and repeated PLS regression using this predictor variable; this process was repeated for 1000 random permutations. We used bootstrapping (resampling with replacement of the 180 ROIs) to calculate the error (SE) in estimating each gene's PLS2 weight. Then we calculated a Z score for each gene using the ratio of the PLS2 weight for that gene to its bootstrap SE. For each gene's Z score, we calculated an FDR corrected p value using a FDR inverse quantile transformation correction to account for winners curse bias (Bigdeli *et al.*, 2016). Genes that survived FDR correction of $q < 0.05$ were included in enrichment analysis. These were ranked according to the PLS2 Z score. Within PLS2, a negative weighting for a specific gene implies that this gene shows lower expression in regions of structural connectivity loss, and a positive weighting implies that the gene shows higher expression; therefore, we assessed genes with negative weightings (downregulated) and positive weightings (upregulated) separately in subsequent analyses.

Supplementary Results

1. Motion and scan quality

To check for differences in motion and image quality between PD low visual performers, PD high visual performers and control subjects that could confound resulting streamlines we calculated three quantitative measures of image quality, derived from each T1-weighted image:

1) Coefficient of joint variation (CJV), between white matter and grey matter. Higher values indicate more head motion and intensity non-uniformity artifacts. (Ganzetti *et al.*, 2016)
2) Entropy focus criterion (EFC). Higher values indicate more ghosting and/or head motion blurring. (Atkinson *et al.*, 1997)
3) Signal to noise ratio (SNR), with higher values indicating better quality image. (Dietrich *et al.*, 2007)

Using a two-sided t-test we found no significant differences in CJV (t=-1.41, p=0.161), EFC (t=-1.01, p=0.313) or SNR (t=1.00, p=0.317) between PD low visual performers and PD high visual performers. We also did not find any statistically significant differences between control participants and PD participants in any of the three metrics. Figure S3 shows each group's image quality metrics.

Figure S3. Image quality metrics in control participants (grey), PD high visual performers (pink) and PD low visual performers (red). CJV: coefficient of joint variation, EFC: Entropy focus criterion, SNR: Signal to noise ratio.

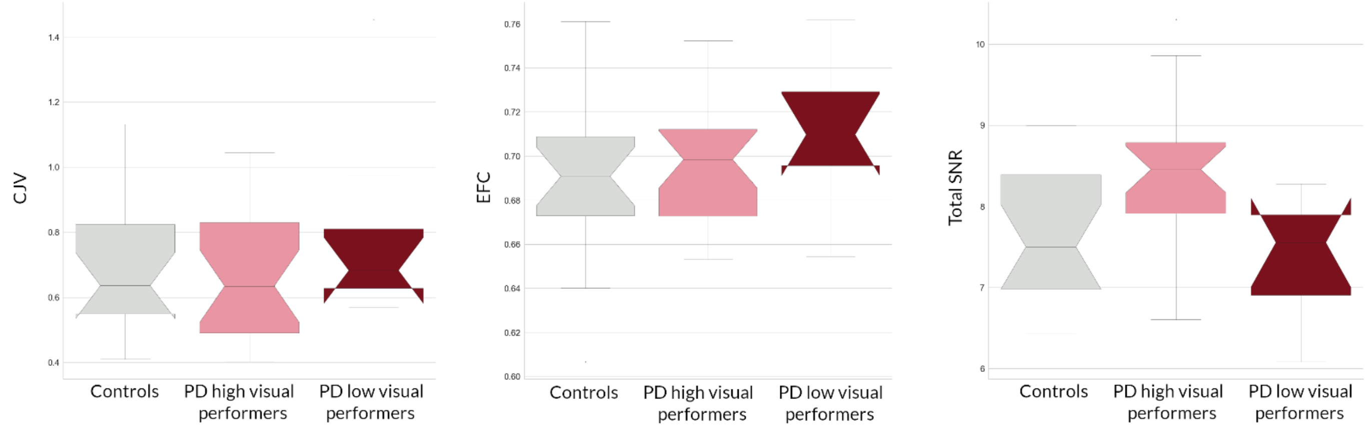

2. Connection length in healthy controls

The average connection length was calculated in healthy controls using the streamline length. The distribution of connection lengths for different connection types, in healthy controls, is seen in Figure S4.

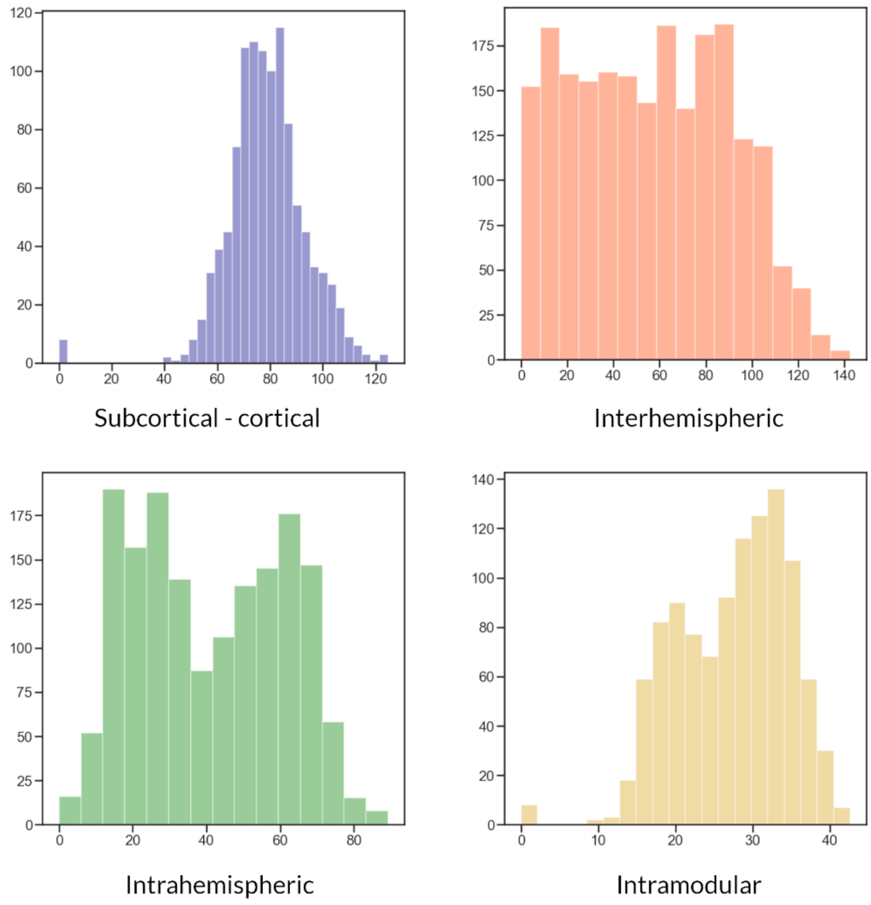

Figure S4. Distribution of connection length across different connection types.

3. Replication analysis: Different module counts

To ensure that the observed differences in connection strength where not influenced by the selected 8 modules, we replicated the analyses with module partition numbers of 6 and 10 using $\gamma = 0.8$ and $\gamma = 1.3$, respectively. Replication analysis using 6 modules showed a similar pattern of loss of connectivity strength in PD low visual performers with interhemispheric connections being primarily affected (77.8% of interhemispheric connections showing reduced strength) followed by the subcortical-cortical connections (16.7% reduced strength) with preserved connectivity in intramodular and intrahemispheric connections. The detailed results of the replication analysis using 6 modules is seen in Figure S5. A similar pattern was seen with module selection of 10 modules: 24% of interhemispheric connections showed reduced connectivity (FDR corrected) in PD low visual performers, with preserved connectivity in the intramodular

and intrahemispheric connections and a trend (not surviving FDR correction) of reduced connectivity in the subcortical-cortical connections (Figure S6).

To ensure that the observed correlation between average connection length in health with loss of connection strength in PD low visual performers were not secondary to module selection of 8 modules, we replicated this analysis using both 6 and 10 modules.

Figure S5. Differences in white matter connection strength in patients with Parkinson's (PD) and low visual performance compared to PD high visual performers, Replication analysis, 6 modules.

The thickness of each connection represents the absolute effect size in PD low visual performers. Red: reduced connectivity strength, Green: Increased connectivity strength, Grey: No statistically significant difference in connectivity strength. * denotes connections that survived FDR correction for multiple comparisons. F: Frontal, M: Motor, V: Occipital.

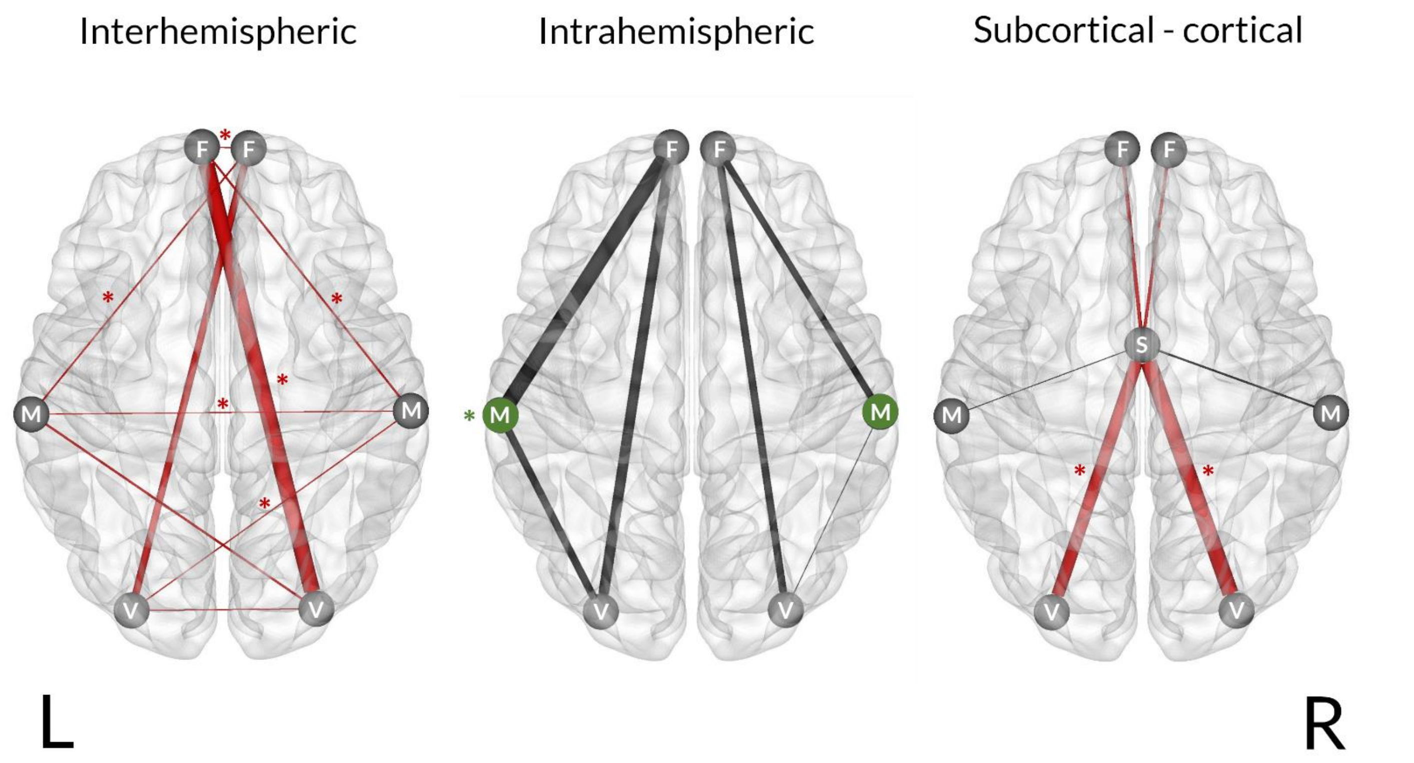

Figure S6. Differences in white matter connection strength in patients with Parkinson's (PD) and low visual performance compared to PD high visual performers, Replication analysis, 10 modules. The thickness of each connection represents the absolute effect size in PD low visual performers. Red: reduced connectivity strength, Green: Increased connectivity strength, Grey: No statistically significant difference in connectivity strength. * denotes connections that survived FDR correction for multiple comparisons. F: Frontal, M: Motor, V: Occipital.

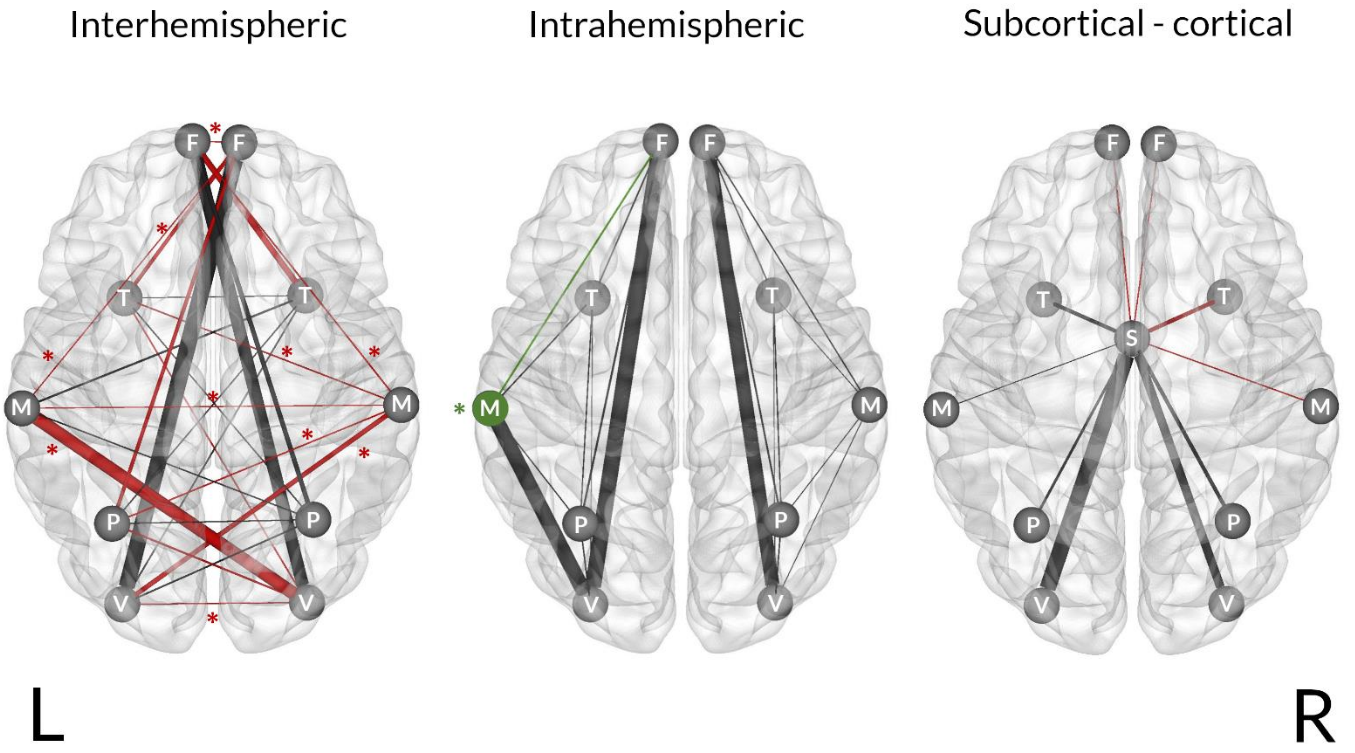

Figure S7. Connection length in controls correlates with loss of connectivity strength in PD low visual

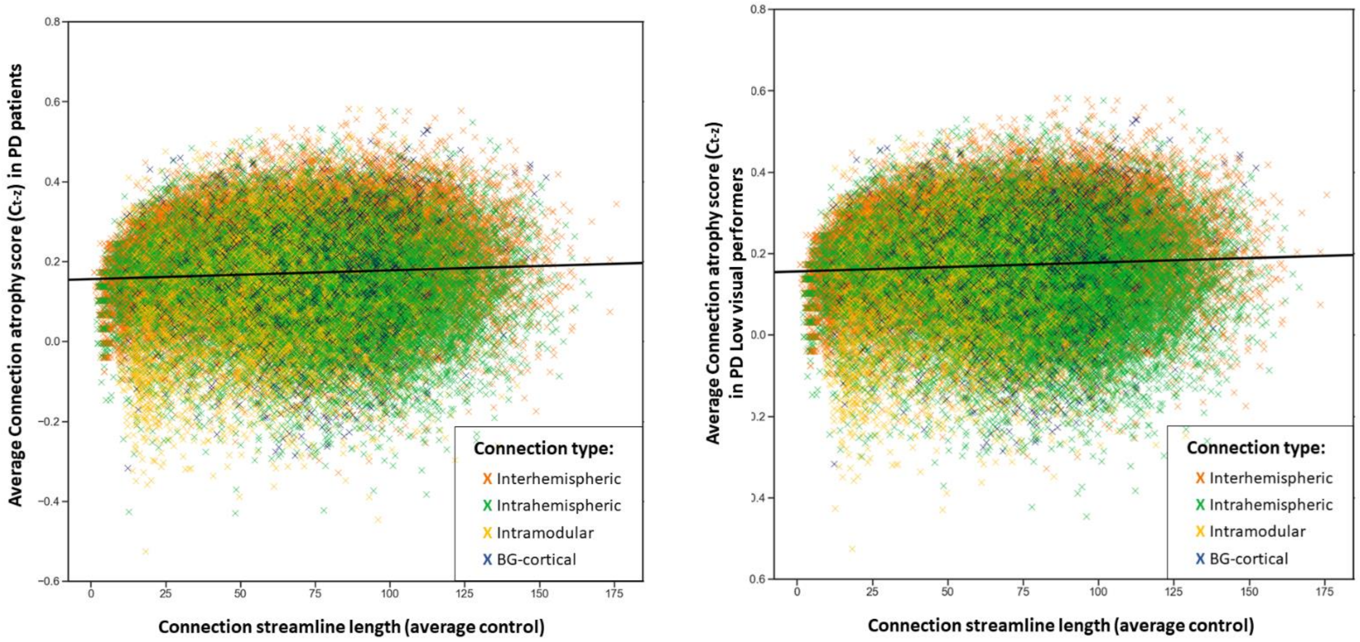

performers. Connections are color-coded according to type. Left: 6 modules, Right: 10 modules

4. Replication analysis: Topological Distance

We replicated our analysis assessing the effect of length on white matter connectivity loss by assessing topological distance (shortest weighted path length), which takes into account not only distance but also the strength of each individual connection. An illustration of shortest weighted path length is seen in Figure S8.A.

Similarly to connection length, different connection types differed in topological distance (df(3, 88000), r^2=0.213, p<0.000): interhemispheric connections were longest, followed by intrahemispheric, intramodular and finally subcortical-cortical connections (Figure S7.B). Subcortical-cortical connections were the longest in streamline length however had the shortest topological distance, likely due to increased connection strength resulting from their increased myelination. Interhemispheric connections were the second longest in terms of topological distance, followed by intrahemispheric connections and finally intramodular connections.

Similar to connection length, topological distance in controls significantly correlated with connection atrophy scores in PD low visual performers (rho=0.082, p<0.000). Interhemispheric connections, which had the higher topological distance in healthy controls, showed the highest atrophy scores, followed by subcortical-cortical connections, whilst intrahemispheric and intramodular connections showed preserved connectivity strength. Correlation between topological distance and atrophy scores were also seen within specific connection types: interhemispheric connections (p<0.001), intrahemispheric (p<0.001) and intramodular connections (p<0.001) with a trend for subcortical-cortical connections (p=0.08).

To ensure that module selection was not influencing these results, these analyses were replicated using 6 and 10 modules (Figure S9).

Figure S8. Topological connection length varies according to connection type and correlates with loss of connectivity strength in PD low visual performers.

A. Illustration of shortest weighted path length between two example nodes A and D. Numbers represent connection weights. When calculating shortest weighted path, length connections are weighted by the inverse of the connection weights, as stronger connections represent shorter paths topologically. Here numbers represent the inverse connection weight, therefore the shortest weighted path is the one highlighted in orange. B. Comparison of shortest weighted path length in controls for different connection types. C. Topological distance (shortest weighted path length) in controls correlates with loss of connectivity strength

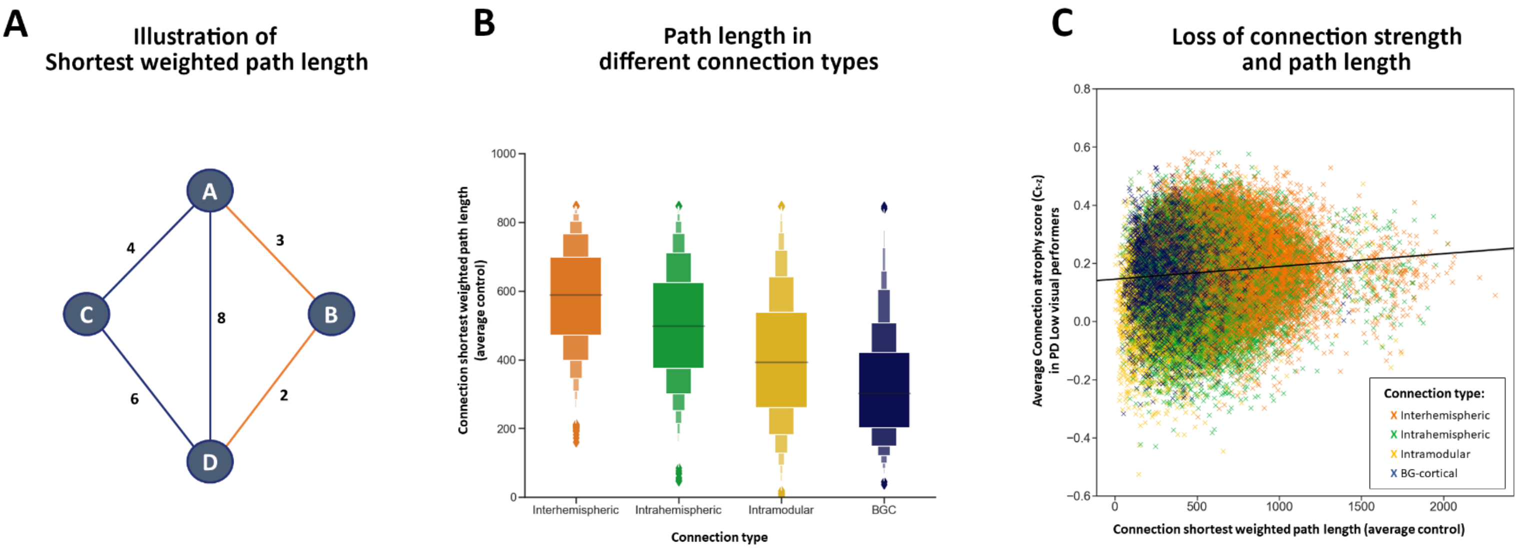

in PD low visual performers.

Figure S9. Correlation between average connection topological distance in healthy controls and loss of

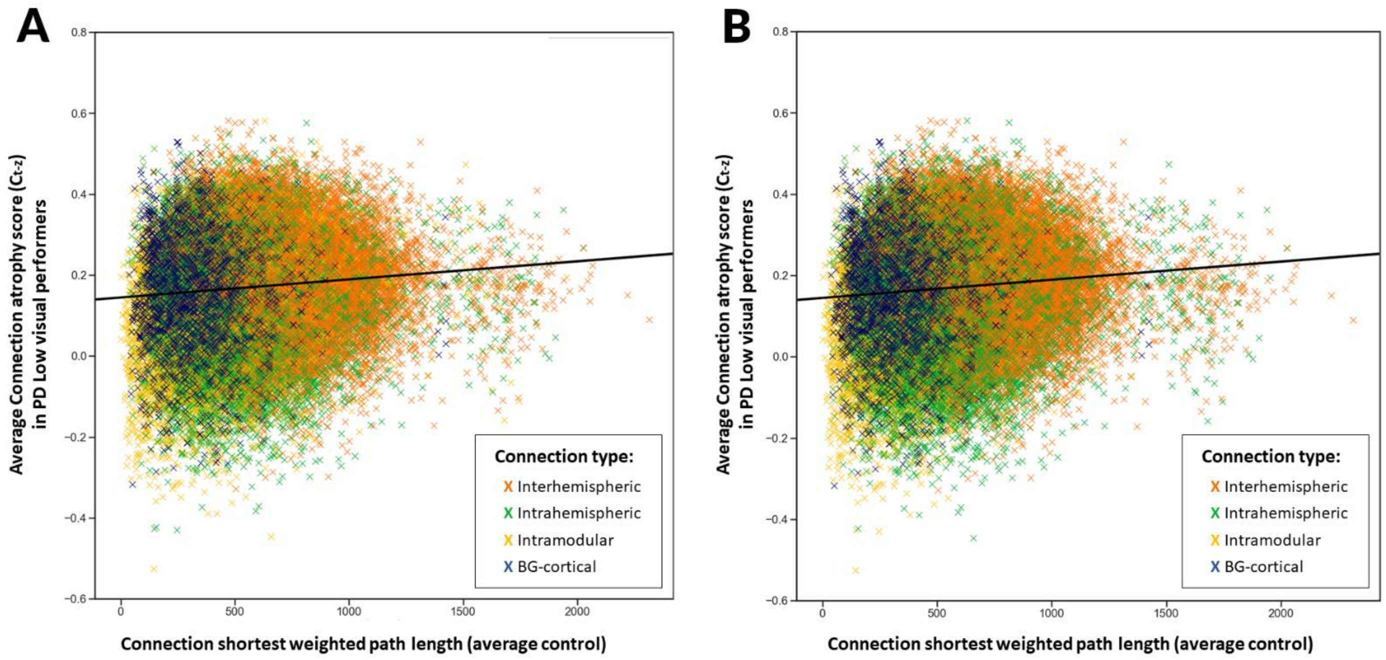

connection strength in PD low visual performers; Replication analysis using 6 modules (A) and 10 modules

5. Replication analysis: Enrichment analysis of random and spatial-spin nulls

We performed additional GO enrichment analyses to mitigate against the possibility of false-positive bias for GO terms in the enrichment analyses of brain-wide transcriptomic data due to null models not accounting for gene-gene co-expression and spatial autocorrelation present within such data (Fulcher *et al.*, 2020). Specifically, we ran GO enrichment analyses for 1000 spatial-spin permutations (using the mean gene weight from these permutations, separately for up-weighted and down-weighted genes) for interhemispheric and subcortical-cortical connections.

For interhemispheric connections, downweighted genes of the spatial-spin null model were most enriched in terms such as regulation of synapse structure or activity, synapse organisation and regulation, dendrite development and cell morphogenesis involved in neuron differentiation (Figure S10A). Upweighted genes were most enriched in terms such as mRNA catabolism, protein targeting, SRP-dependent cotranslational protein targeting to membrane, viral transcription and mitochondrion organisation (Figure S10B). These were significantly divergent from the results of our main analysis.

For subcortical-cortical connections, the most enriched terms for downweighted genes of the spatial-spin null model were electron transport chain, oxidation-reduction process, small molecule metabolism, generation of precursor metabolites and energy and mitochondrion organisation (Figure S10C). The most enriched terms for upweighted genes were RNA metabolism, splicing and biosynthesis, regulation of RNA metabolism and regulation of nucleobase-containing compound metabolism (Figure S10D).

The full GO terms for the two spatial-spin null models can be seen in Supplementary Table S6.

Figure S10. Divergent enrichment patterns in spatial-spin null models for both interhemispheric and subcortical-cortical connections.

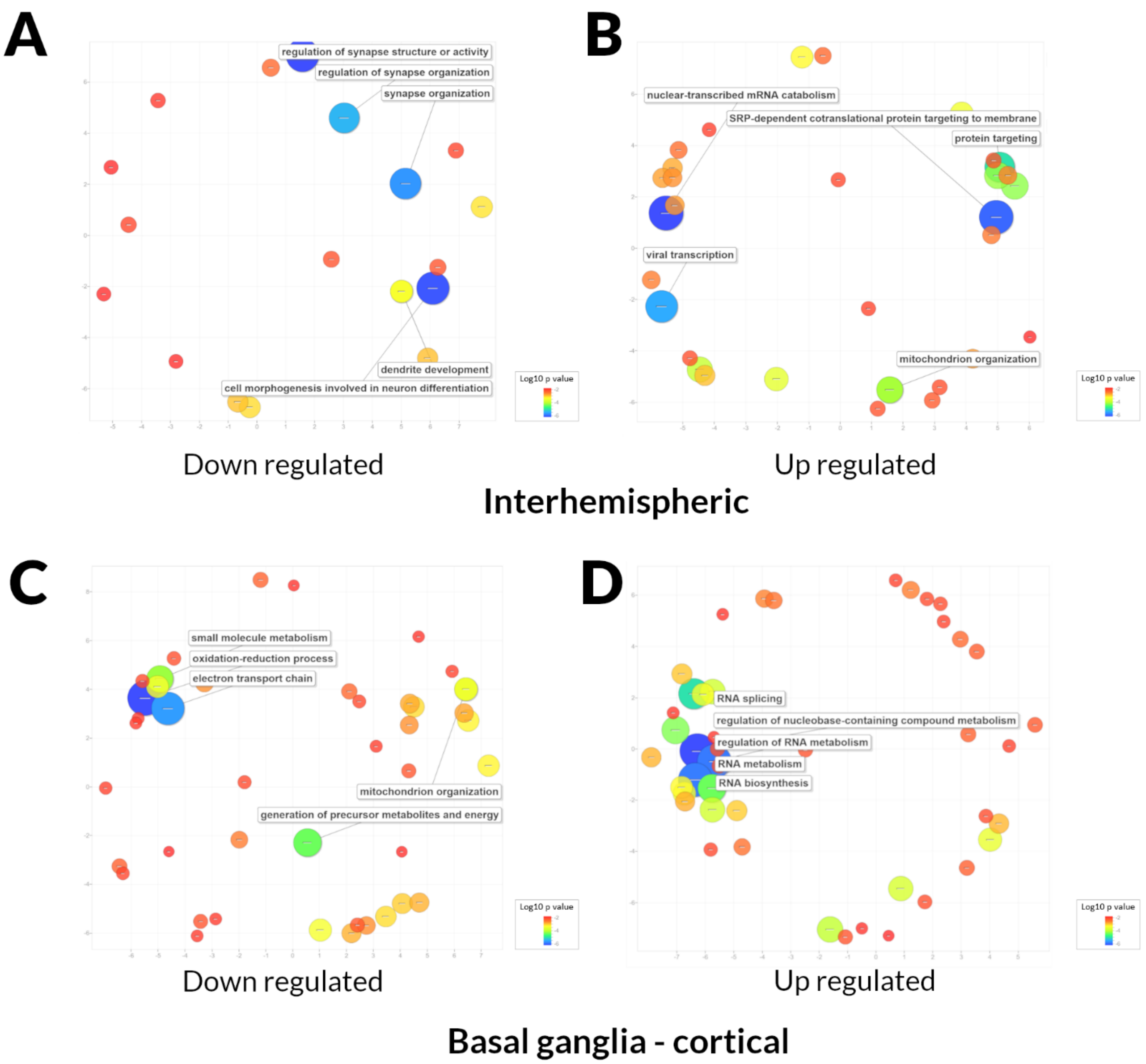

6. Replication analysis: EWCE with different single cell transcription dataset

To ensure that our results regarding differential cell type enrichment in interhemispheric and subcortical-cortical connections were not dependent on the dataset used, we replicated our EWCE analysis using a different human-derived single nuclear RNA-sequencing dataset from the Regev group (Habib *et al.*, 2017) containing cell-type specific gene expression from the hippocampus and the prefrontal cortex. Correction for multiple comparisons was performed using the Benjamini-Hochberg method.

Similar pattern of cell type enrichment was seen for the replications dataset as had been found the AIBS dataset (Figure S11). For subcortical-cortical connections downweighted genes were enriched in oligodendrocytes and upweighted genes were enriched in neurons and GABAergic interneurons (Figure S9). As we had previously found using the AIBS dataset, a different pattern of cell type enrichment was seen for interhemispheric connections, with downweighted genes significantly enriched for some neuronal types and upweighted genes were not significantly enriched in any cell type (Figure S11).

Additionally, we performed EWCE analysis using different gene cut-offs in both datasets for both connection types to ensure that our results were not influenced by the somewhat arbitrary cut-off of 20% (using gene lists comprised of the top 20% downweighted and upweighted genes for subcortical-cortical and interhemispheric connection types). Overall, we saw qualitatively similar results using gene lists comprised of the top 10% significantly upweighted and downweighted genes (Figure S12), the top 30% of genes (Figure S13) and the top 50% of genes (Figure S14).

Figure S11. Stable cell type enrichment patterns of the top 20% most highly downweighted and upweighted genes in interhemispheric and subcortical-cortical connections in PD low visual performers using the Regev dataset. Results presented as standard deviations from the mean; * statistically significant results. ASC: Astrocytes, END: Endothelial cells, NSC: Neuronal stem cells, OPC: Oligodendrocyte Precursor cells, MC: Microglia, exCA: Pyramidal CA, exPFC: Pyramidal prefrontal cortex, exDG: Granule neurons hippocampus dentate gyrus, GABA: GABAergic interneurons, ODC: Oligodendrocytes

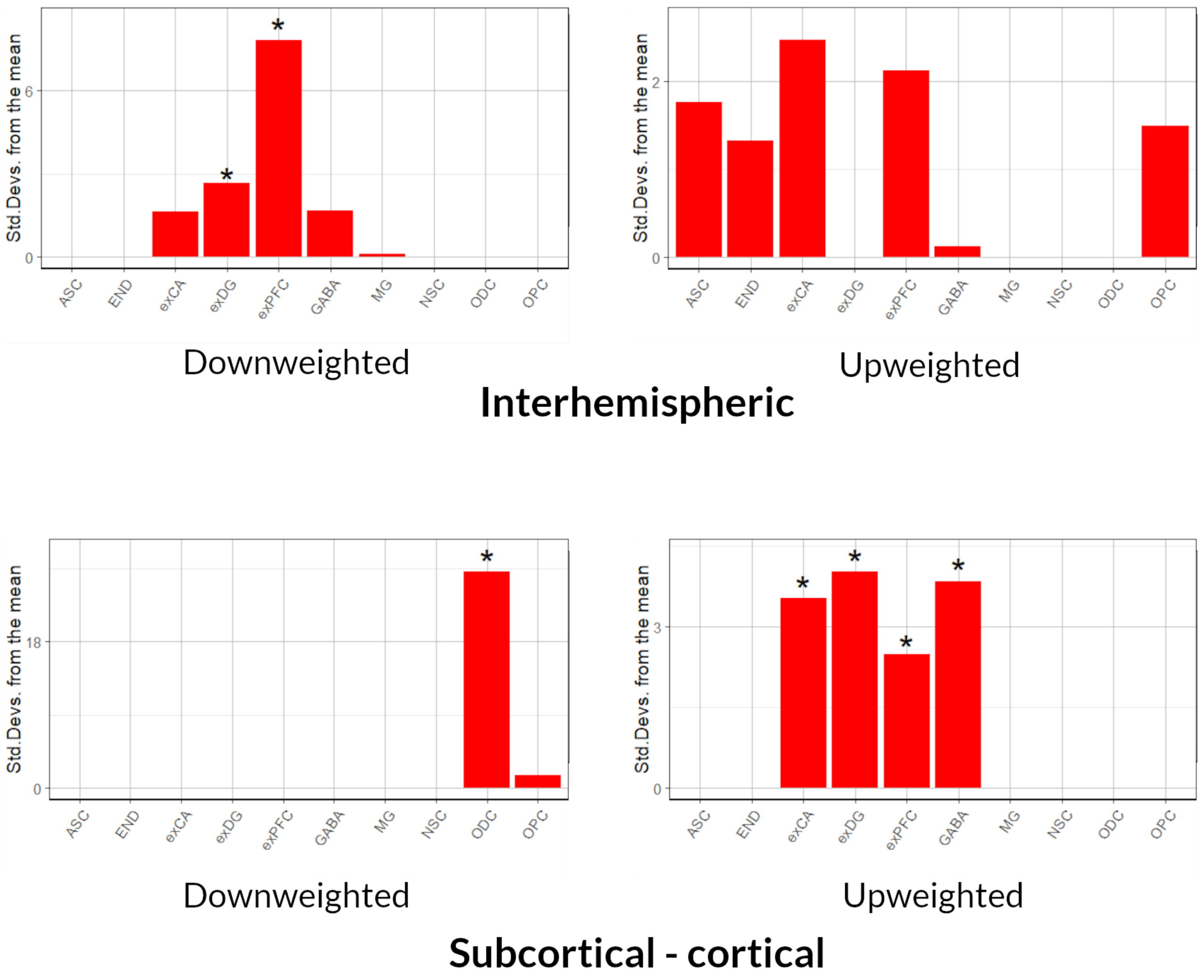

Figure S12. Stable cell type enrichment patterns in the top 10% most weighted genes for interhemispheric and subcortical-cortical connections in PD low visual performers.

A. AIBS dataset. B. Regev dataset.

Results presented as standard deviations from the mean; * statistically significant results. ASC: Astrocytes, END: Endothelial cells, NSC: Neuronal stem cells, OPC: Oligodendrocyte Precursor cells, MC: Microglia, exCA: Pyramidal CA, exPFC: Pyramidal prefrontal cortex, exDG: Granule neurons hippocampus dentate

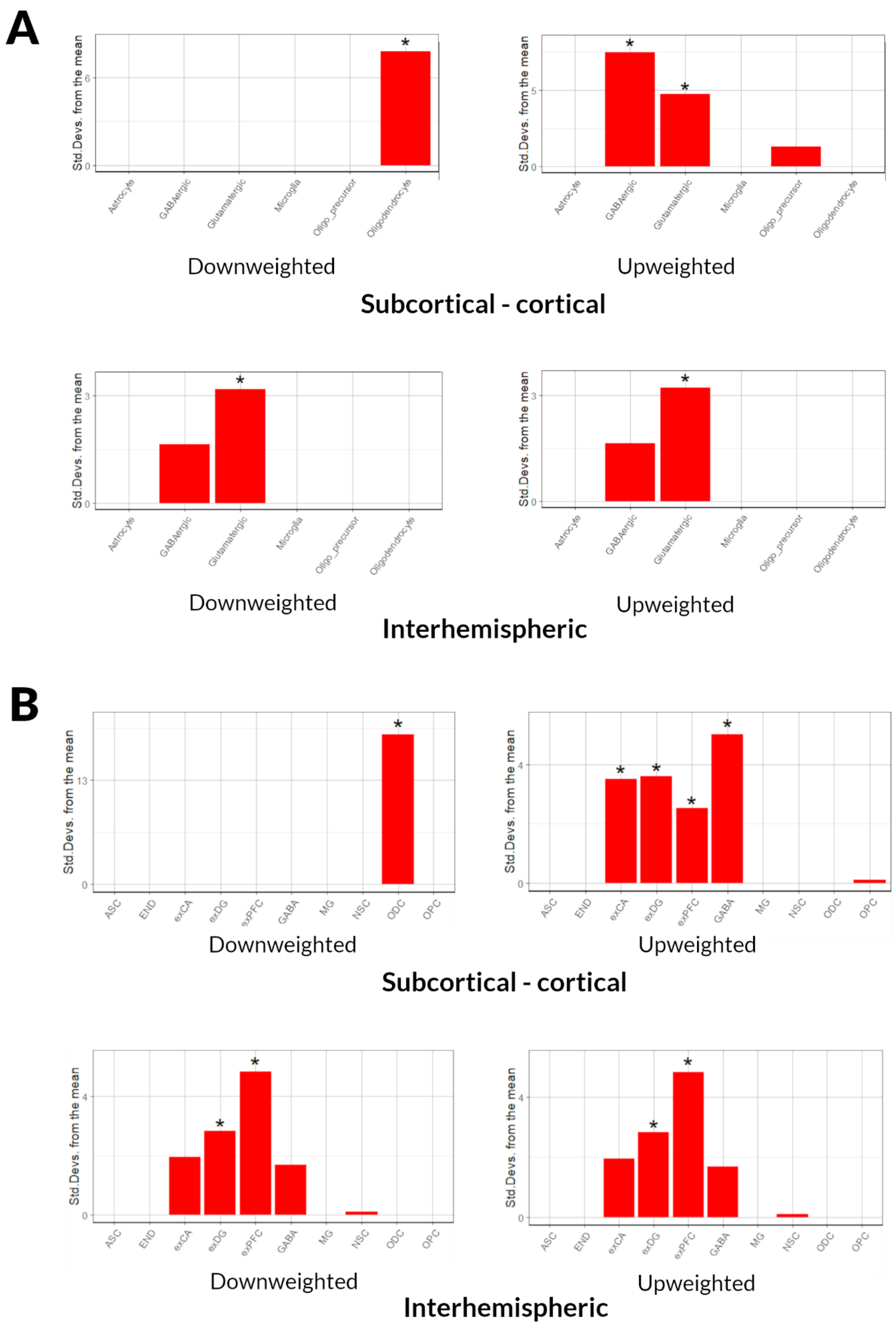

gyrus, GABA: GABAergic interneurons, ODC: Oligodendrocytes

Figure S13. Stable cell type enrichment patterns in the top 30% most weighted genes for interhemispheric and subcortical-cortical connections in PD low visual performers.

A. AIBS dataset. B. Regev dataset.

Results presented as standard deviations from the mean; * statistically significant results. ASC: Astrocytes, END: Endothelial cells, NSC: Neuronal stem cells, OPC: Oligodendrocyte Precursor cells, MC: Microglia, exCA: Pyramidal CA, exPFC: Pyramidal prefrontal cortex, exDG: Granule neurons hippocampus dentate gyrus, GABA: GABAergic interneurons, ODC: Oligodendrocytes

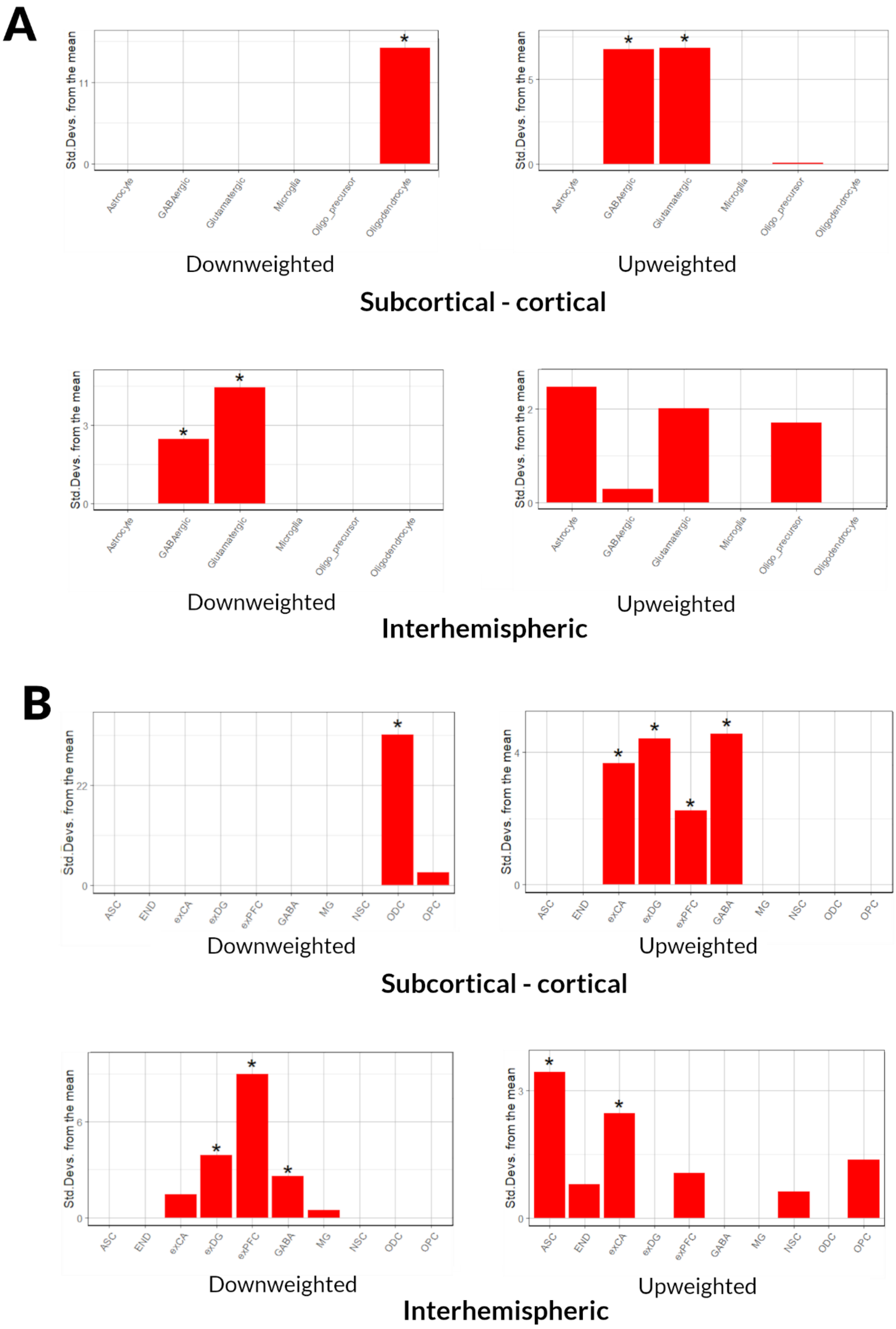

Figure S14. Stable cell type enrichment patterns in the top 50% most weighted genes for interhemispheric and subcortical-cortical connections in PD low visual performers.

A. AIBS dataset. B. Regev dataset.

Results presented as standard deviations from the mean; * statistically significant results. ASC: Astrocytes, END: Endothelial cells, NSC: Neuronal stem cells, OPC: Oligodendrocyte Precursor cells, MC: Microglia, exCA: Pyramidal CA, exPFC: Pyramidal prefrontal cortex, exDG: Granule neurons hippocampus dentate gyrus, GABA: GABAergic interneurons, ODC: Oligodendrocytes

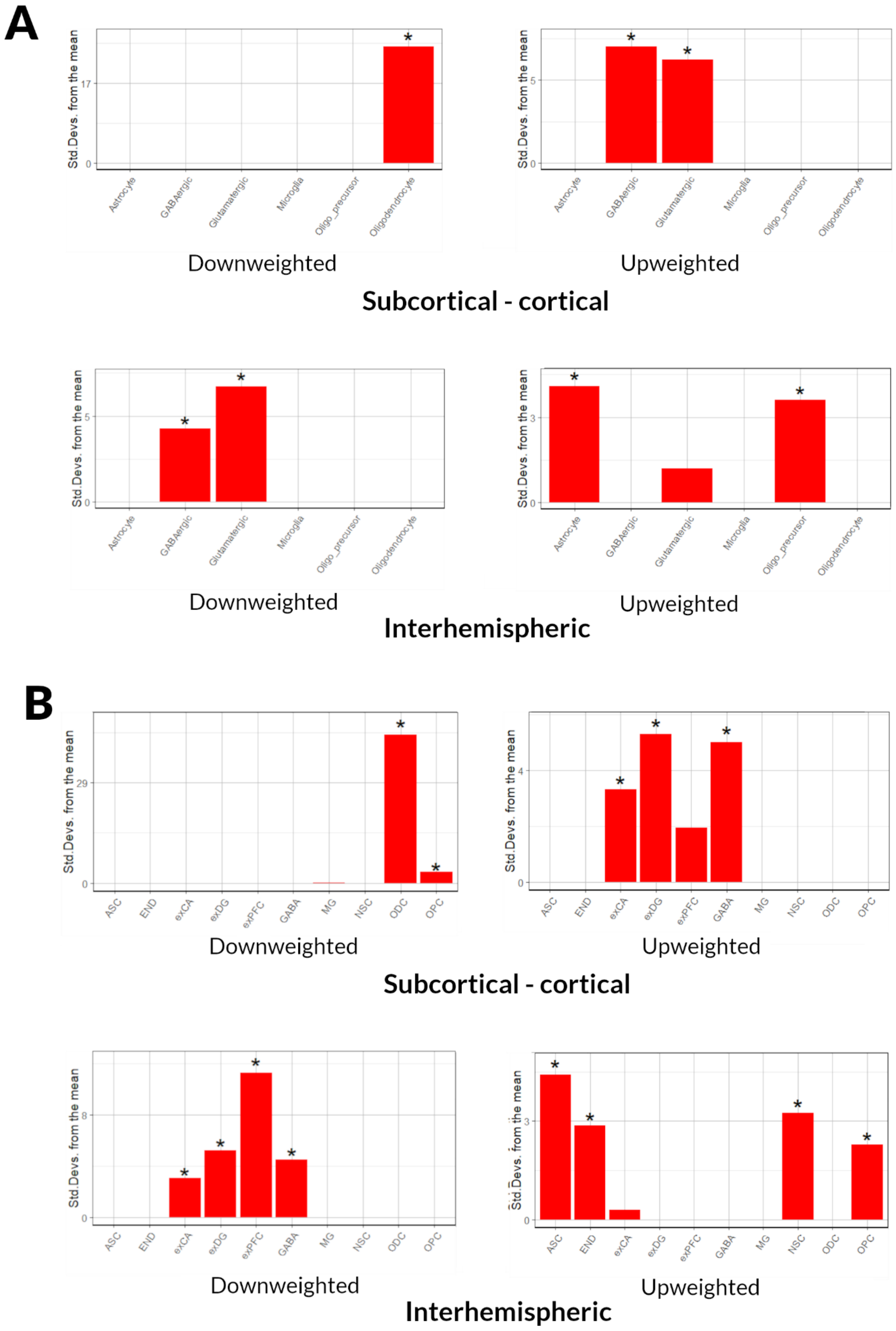

6. Table S1. Genes with common variations resulting to increased risk for Parkinson's disease overlapping with upweighted genes for subcortical-cortical connections

Gene Name
PAM
INPP5F
MIPOL1
CRLS1
SATB1
LRRK2
C5orf24
WNT3
MAP4K4
ITPKB
STK39
HIP1R
RNF141
CRHR1
BAG3

Additional Files

Filename	Description
**TableS2_ModuleAllocations.pdf**	**Table S2.** Regions allocated to each module for 8, 6 and 10 module partitions.
**TableS3_PLSWeights_Genes.xlsx**	**Table S3**. Individual gene weights from the second PLS component for interhemispheric and subcortical-cortical connections.
**TableS4_PLSWeights_Regions.xlsx**	**Table S4**. Individual ROI weights from the second PLS component for interhemispheric and subcortical-cortical connections.
**TableS5_FullGOterms.csv**	**Table S5.** Significantly enriched GO terms for biological processes in up- and downweighted genes associated with interhemispheric and subcortical-cortical connections.
**TableS6_FullGOtermsNullModels.csv**	**Table S6.** Significantly enriched GO terms for biological processes in up- and downweighted genes from 1000 spatial-spin permutations.

URLs:

MRtrix3.0 https://www.mrtrix.org/
Allen human brain atlas: https://human.brain-map.org/
Pre-processed expression data: https://figshare.com/articles/AHBAdata/6852911
g:Profiler https://biit.cs.ut.ee/gprofiler/gost
Reduce and Visualise Gene Ontology tool http://revigo.irb.hr/
PLS, hoggorm library: https://github.com/olivertomic/hoggorm
EWCE: https://github.com/NathanSkene/EWCE
AIBS single cell RNA dataset https://portal.brain-map.org/atlases-and-data/rnaseq
Hypergeometric overlap: https://github.com/brentp/bio-playground/blob/master/utils/list_overlap_p.py

References

Alexander-Bloch A, Shou H, Liu S, Satterthwaite TD, Glahn DC, Shinohara RT, et al. On testing for spatial correspondence between maps of human brain structure and function. Neuroimage 2018; 178: 540–51.

Atkinson D, Hill DL, Stoyle PN, Summers PE, Keevil SF. Automatic correction of motion artifacts in magnetic resonance images using an entropy focus criterion. IEEE Trans Med Imaging 1997; 16: 903–10.

Bigdeli TB, Lee D, Webb BT, Riley BP, Vladimirov VI, Fanous AH, et al. A simple yet accurate correction for winner's curse can predict signals discovered in much larger genome scans. Bioinformatics 2016; 32: 2598–603.

Bullmore E, Sporns O. Complex brain networks: graph theoretical analysis of structural and functional systems. Nat Rev Neurosci 2009; 10: 186–98.

Dietrich O, Raya JG, Reeder SB, Reiser MF, Schoenberg SO. Measurement of signal-to-noise ratios in MR images: Influence of multichannel coils, parallel imaging, and reconstruction filters. Journal of Magnetic Resonance Imaging 2007; 26: 375–85.

Dijkstra EW. A note on two problems in connexion with graphs. Numer Math 1959; 1: 269–71.

Fulcher BD, Arnatkevičiūtė A, Fornito A. Overcoming bias in gene-set enrichment analyses of brain-wide transcriptomic data. bioRxiv 2020; (preprint): 2020.04.24.058958.

Ganzetti M, Wenderoth N, Mantini D. Intensity Inhomogeneity Correction of Structural MR Images: A Data-Driven Approach to Define Input Algorithm Parameters [Internet]. Front Neuroinform 2016; 10[cited 2019 Dec 19] Available from: https://www.frontiersin.org/articles/10.3389/fninf.2016.00010/full

Habib N, Avraham-Davidi I, Basu A, Burks T, Shekhar K, Hofree M, et al. Massively parallel single-nucleus RNA-seq with DroNc-seq. Nat Methods 2017; 14: 955–8.

McColgan P, Gregory S, Seunarine KK, Razi A, Papoutsi M, Johnson E, et al. Brain Regions Showing White Matter Loss in Huntington's Disease Are Enriched for Synaptic and Metabolic Genes. Biol Psychiatry 2018; 83: 456–65.

Morgan SE, Seidlitz J, Whitaker KJ, Romero-Garcia R, Clifton NE, Scarpazza C, et al. Cortical patterning of abnormal morphometric similarity in psychosis is associated with brain expression of schizophrenia-related genes. Proceedings of the National Academy of Sciences of the United States of America 2019; 116: 9604–9.

Romero-Garcia R, Warrier V, Bullmore ET, Baron-Cohen S, Bethlehem RAI. Synaptic and transcriptionally downregulated genes are associated with cortical thickness differences in autism. Mol Psychiatry 2019; 24: 1053–64.

Rosipal R, Krämer N. Overview and recent advances in partial least squares. Lecture Notes in Computer Science (including subseries Lecture Notes in Artificial Intelligence and Lecture Notes in Bioinformatics) 2006; 3940 LNCS: 34–51.

Rubinov M, Ypma RJF, Watson C, Bullmore ET. Wiring cost and topological participation of the mouse brain connectome. Proc Natl Acad Sci U S A 2015; 112: 10032–7.

Vértes PE, Rittman T, Whitaker KJ, Romero-Garcia R, Váša F, Kitzbichler MG, et al. Gene transcription profiles associated with inter-modular hubs and connection distance in human functional magnetic resonance imaging networks. Philos Trans R Soc Lond, B, Biol Sci 2016; 371

Whitaker KJ, Vértes PE, Romero-Garcia R, Váša F, Moutoussis M, Prabhu G, et al. Adolescence is associated with genomically patterned consolidation of the hubs of the human brain connectome. Proc Natl Acad Sci USA 2016; 113: 9105–10.
